# Maternal and Fetal Genetic Associations of PTGER3 and PON1 with Preterm Birth

**DOI:** 10.1371/journal.pone.0009040

**Published:** 2010-02-03

**Authors:** Kelli K. Ryckman, Nils-Halvdan Morken, Marquitta J. White, Digna R. Velez, Ramkumar Menon, Stephen J. Fortunato, Per Magnus, Scott M. Williams, Bo Jacobsson

**Affiliations:** 1 Department of Molecular Physiology and Biophysics and Center for Human Genetics Research, Vanderbilt University, Nashville, Tennessee, United States of America; 2 Department of Obstetrics and Gynecology, Haukeland University Hospital, Bergen, Norway; 3 Norwegian Institute of Public Health, Oslo, Norway; 4 Dr. John T. Macdonald Foundation Department of Human Genetics and Miami Institute of Human Genomics, Miller School of Medicine, University of Miami, Miami, Florida, United States of America; 5 The Perinatal Research Center, Nashville, Tennessee, United States of America; 6 Department of Epidemiology, Emory University, Atlanta, Georgia, United States of America; 7 Perinatal Center, Department of Obstetrics and Gynecology, Institute of Clinical Sciences, Sahlgrenska Academy, Gothenburg, Sweden; 8 Department of Obstetrics and Gynecology, Rikshospitalet, Oslo, Norway; Instituto Gulbenkian de Ciência, Portugal

## Abstract

**Objective:**

The purpose of this study was to identify associations between maternal and fetal genetic variants in candidate genes and spontaneous preterm birth (PTB) in a Norwegian population and to determine the effect size of those associations that corroborate a previous study of PTB.

**Methods:**

DNA from 434 mother-baby dyads (214 cases and 220 controls) collected from the Norwegian Mother and Child Cohort (MoBa) was examined for association between 1,430 single nucleotide polymorphisms in 143 genes and PTB. These results were compared to a previous study on European Americans (EA) from Centennial Women's Hospital in Nashville, TN, USA. Odds ratios for SNPs that corroborated the Cenntennial study were determined on the combined MoBa and Centennial studies.

**Results:**

In maternal samples the strongest results that corroborated the Centennial study were in the prostaglandin E receptor 3 gene (PTGER3; rs977214) (combined genotype p = 3×10^−4^). The best model for rs977214 was the AG/GG genotypes relative to the AA genotype and resulted in an OR of 0.55 (95% CI = 0.37–0.82, p = 0.003), indicating a protective effect. In fetal samples the most significant association in the combined data was rs854552 in the paraoxonase 1 gene (PON1) (combined allele p = 8×10^−4^). The best model was the TT genotype relative to the CC/CT genotypes, and resulted in an OR of 1.32 (95% CI = 1.13–1.53, p = 4×10^−4^).

**Conclusions:**

These studies identify single locus associations with preterm birth for both maternal and fetal genotypes in two populations of European ancestry.

## Introduction

Preterm birth (PTB) (<37 weeks of gestation) is a major obstetrical problem, contributing to a substantial proportion of infant morbidity and mortality worldwide. Preterm birth accounts for as much as 75% of perinatal mortality and a disproportionately large percentage of infant morbidities [1]–[4]. In the US alone, of the approximately five million births each year almost 13% are born preterm, accounting for greater than $26 billion per year in excess health care costs [5], [6]. The majority of PTB (∼75%) results from spontaneous contractions often associated with infection, prelabor preterm rupture of the membranes and unknown causes [7]. Based on human and animal studies, four main pathways have been hypothesized to lead to PTB: 1) activation of maternal or fetal hypothalamic pituitary-adrenal axis, 2) inflammation and infection, 3) decidual hemorrhage, and 4) uterine distension [8]. These pathways converge on a final terminal pathway where uterotonins, such as prostaglandins and extra cellular matrix (ECM) degrading proteases, are released leading to early contractions, cervical ripening and rupture of the membranes eventually resulting in PTB. The specific factors involved in these putative PTB pathways and how each contributes to the etiology of PTB are poorly understood.

There is growing evidence that among the risk factors that have been hypothesized to be associated with PTB genetic predisposition plays a significant role. First, personal and family history of PTB are among the strongest known risk factors for PTB [9]–[11]. Second, twin studies estimate the heritability of PTB at 20–40% [12], [13]. In addition, there is substantial evidence suggesting geographic ancestry is associated with risk for PTB; therefore, it is reasonable to conclude that genetic variations can affect risk [14]–[16]. Finally, in the last ten years many studies have found significant genetic associations with PTB, particularly with genes in the inflammation and infection pathway [17]–[19]. However, often these findings fail to replicate [20].

Recently, a candidate gene association study of PTB was performed examining 1,536 single nucleotide polymorphisms (SNPs) in approximately 130 candidate genes [21]. The strongest maternal associations with PTB were in factor V (FV), factor VII (FVII) and tissue plasminogen activator (tPA) in European Americans (EA). These genes are involved in the complement-coagulation pathway, related to decidual hemorrhage. The most significant fetal associations were in interleukin 10 receptor antagonist (IL-10RA) and other genes related to the infection and inflammation pathway.

In the present study we sought to identify genetic risk factors for PTB in a Norwegian cohort, and corroborate these results with data from the study described above[21]. In addition to the SNPs that were previously studied we included 132 SNPs from 14 genes emphasizing genes from the complement coagulation pathway that were not previously represented because of strong associations between SNPs in this pathway and PTB previously identified in EA. The results of the two studies were compared to identify genes involved in PTB pathways.

## Results

### Demographic and Clinical Characteristics

Clinical differences between cases and controls for the Norwegian Mother and Child Cohort (MoBa) are presented in Table 1. There were no statistically significant differences between cases and controls for APGAR score at either 1 minute or 5 minutes, maternal age or smoking; however, as expected, cases had babies with lower birth weight and gestational age (p<0.001 for both). Additionally, cases and controls significantly differed in parity (p<0.001), where having one or more children was protective against PTB (OR = 0.45, CI = 0.30–0.67, p-value<0.001).

**Table 1:** Clinical characteristics.

Variable	1Cases (n = 207)	Controls (n = 217)	2p
Parity	0 [0–4]	1 [0–4]	<0.001
Gestational Age (days)	253 [182–258]	280 [273–286]	<0.001
Birth weight (grams)	2810 [950–4000]	3650 [2610–4970]	<0.001
APGAR at 1 minute (%<7)	16 (8%)	12 (6%)	0.355
APGAR at 5 minutes (%<7)	3 (1%)	5 (2%)	0.522
Maternal Age (yrs)	29 [20]–[34]	30 [21]–[34]	0.203
Smoking (%)	22 (12%)	21 (11%)	0.770

Parity is defined as the number of times a woman has given birth.

Medians are reported with the range in brackets.

1 Cases are defined as <37 weeks gestation compared with the women delivering at term between 39–40 weeks (controls).

2 P-values are calculated by Mann Whitney U- test for continuous variables and chi-square test or Fisher's exact test for dichotomous variables.

The MoBa and the Centennial Medical Center study (Cenn) used slightly different but substantially overlapping gestational age definition; in MoBa <36^6/7^ weeks was used and in the Cenn study cases were defined by a gestational age of <36^0/7^ weeks (Table 2). The controls were defined by a gestational age of ≥39^0/7^ weeks in the Moba study and ≥37^0/7^ weeks in the Cenn study. Gravidity, gestational age and birth weight differed between the two study populations in cases and controls. When data from the two studies are combined, the median gestational age was 248 days (range = 166–258) in cases and 278 days in controls (range = 257–296).

**Table 2:** Comparisons of clinical variables between MoBa and Cenn.

Population	Variable	MoBa (n = 217)	Cenn (n = 199)	1p
Controls	Gravidity	1 [0–7]	2 [1]–[8]	<0.0001
	Gestational Age (days)	280 [273–286]	274 [257–296]	<0.0001
	Birthweight (grams)	3650 [2610–4970]	3446 [2100–4661]	<0.0001
	APGAR at 1 minute (% <7)	12 (6%)	4 (2%)	0.081
	APGAR at 5 minutes (% <7)	5 (2%)	0 (0%)	0.037
	Maternal Age (yrs)	30 [21]–[34]	28 [16]–[43]	0.045
	Smoking (%)	21 (11%)	28 (15%)	0.284
Cases	Gravidity	0 [0–5]	2 [1]–[9]	<0.0001
	Gestational Age (days)	253 [182–258]	239 [166–255]	<0.0001
	Birthweight (grams)	2810 [950–4000]	2150 [370–3790]	<0.0001
	APGAR at 1 minute (% <7)	16 (8%)	42 (25%)	<0.001
	APGAR at 5 minutes (% <7)	3 (1%)	10 (6%)	0.018
	Maternal Age (yrs)	29 [20]–[34]	27 [17]–[40]	0.065
	Smoking (%)	22 (12%)	54 (32%)	<0.001

Gravidity is defined as the number of times a woman has been pregnant.

Medians are reported with the range in brackets.

1 P-values are calculated by Mann Whitney U- test for continuous variables and chi-square test for dichotomous variables.

### Single Locus Association

**Figure 1:**
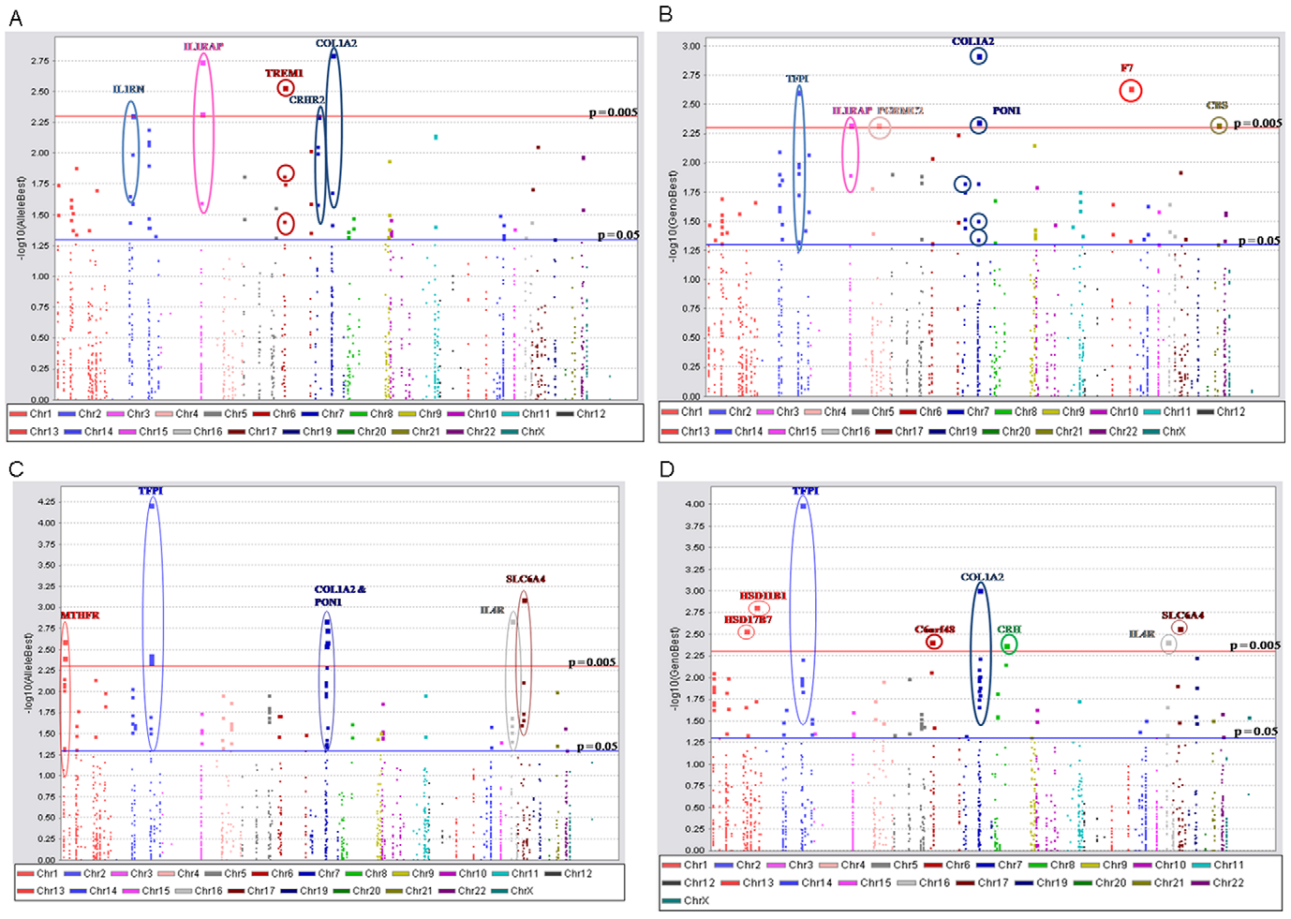
Single locus association results in MoBa study. Each point on the graph represents an association test either allelic (a, c) or genotypic (b, d). The x axis is SNP position in chromosomal order and the y axis is the inverse negative log of the p-value. Suggestive associations are highlighted in genes with at least one association at p<0.005. TFPI (rs6434222) is significant after correction for multiple testing with FDR. Maternal samples are represented in panels a and b, and fetal samples are represented in panels c and d.

Of the 1430 SNPs analyzed in MoBa, there were 125 suggestive (p<0.05) allelic and/or genotypic single locus results in maternal samples and 142 suggestive allelic and/or genotypic results in fetal samples (Figure 1A-D, Table S3). The most significant maternal single locus result was in collagen, type 1, alpha 2 (COL1A2) at rs2472 (allelic p = 2×10^−3^, genotypic p = 1×10^−3^) (Table 3). This SNP was also associated in fetal samples (allelic p = 8×10^−3^, genotypic p = 8×10^−3^) (Table 3). In maternal samples this SNP had an OR of 0.32 (95% CI = 0.16–0.66, p = 2×10^−3^) when comparing the AG genotype to the AA genotype. This result remained significant after adjusting for parity (p = 2×10^−3^), and was also identified using the method PRAT (Table 3). In addition to rs2472, nine SNPs of the thirty-four examined in COL1A2 associated with PTB (p<0.05); seven SNPs associated in fetal samples only, one in maternal samples only and one in both maternal and fetal samples (Table 3). None of these results were significant after correction for multiple testing with FDR (q = 0.20).

**Table 3:** Significant single locus maternal and fetal associations.

Gene	SNP	Genic Role	Sample	Allele	Case MAF	Control MAF	Allele p	Genotype p	PRAT Case	PRAT Control
COL1A2	rs388625	Intron	Fetal	A	0.37	0.46	0.01	0.01	8×10^−3^	0.05
	rs411717	Intron	Fetal	A	0.37	0.46	0.01	0.01	8×10^−3^	0.05
	rs420257	Intron	Fetal	G	0.24	0.34	1×10^−3^	1×10^−3^	2×10^−3^	0.03
	rs389328	Intron(boundary)	Fetal	T	0.12	0.19	0.01	0.01	0.02	0.12
	rs42524	Coding exon	Fetal	G	0.19	0.26	0.01	0.02	0.02	0.32
	rs2521205	Intron	Maternal	C	0.43	0.51	0.02	0.05	0.03	0.654
			Fetal	C	0.45	0.52	0.04	0.13	0.06	0.83
	rs42528	Intron	Fetal	A	0.20	0.28	8×10^−3^	0.02	0.01	0.54
	rs2472	Intron	Maternal	G	0.03	0.07	2×10^−3^	1×10^−3^	0.02	<1×10^−3^
			Fetal	G	0.03	0.08	8×10^−3^	8×10^−3^	0.04	2×10^−3^
	rs441051	Intron	Fetal	A	0.17	0.25	3×10^−3^	0.01	4×10^−3^	0.59
	rs7804898	Intron	Maternal	G	10.17	0.14	0.21	0.02	0.04	0.05
TFPI	rs12693471	Downstream	Maternal	G	0.27	0.35	9×10^−3^	0.01	0.01	0.84
			Fetal	G	0.26	0.36	4×10^−3^	0.01	4×10^−3^	0.68
	rs8176541	Intron	Maternal	A	0.27	0.35	8×10^−3^	0.01	0.01	0.78
			Fetal	A	0.26	0.36	4×10^−3^	0.01	4×10^−3^	0.66
	rs7586970	Intron	Maternal	G	0.27	0.35	8×10^−3^	0.01	0.01	0.78
			Fetal	G	0.26	0.36	4×10^−3^	0.01	4×10^−3^	0.65
	rs3213739	Intron	Maternal	A	10.38	0.46	0.03	2×10^−3^	9×10^−3^	0.12
			Fetal	A	0.38	0.48	4×10^−3^	0.01	1×10^−3^	0.69
	rs8176508	Intron	Maternal	A	0.41	0.36	0.16	0.05	0.09	0.03
			Fetal	A	0.41	0.32	4×10^−3^	0.01	2×10^−3^	0.31
	rs2041778	Intron	Maternal	G	0.33	0.40	0.04	0.06	0.09	0.03
			Fetal	G	0.32	0.39	0.03	0.08	0.03	0.59
	rs3755248	Intron	Maternal	G	0.27	0.35	7×10^−3^	0.02	0.01	0.84
			Fetal	G	0.28	0.34	0.03	0.09	0.05	0.81
	rs7573488	Intron	Maternal	G	10.24	0.31	0.01	0.01	9×10^−3^	0.72
			Fetal	G	0.23	0.31	0.02	0.06	0.03	0.37
	rs6434222	Intron	Fetal	T	0.17	0.08	6×10^−5^ *	1×10^−4^ *	<1×10^−3^	0.63

*Significant after correction for multiple testing with FDR (q = 0.2).

1 Cases deviated from HWE in maternal samples at rs3213739 (p = 0.01), rs7573488 (p = 0.04), rs7804898 (p = 0.05).

The most significant fetal single locus result was in tissue factor pathway inhibitor (TFPI) at rs6434222 (allelic p = 6×10^−5^, genotypic p = 1×10^−4^, both of which were significant after FDR correction) (Table 3). This SNP was not significantly associated with PTB in maternal samples. In fetal samples this SNP had an OR of 2.49 (95% CI = 1.59–3.91, p = 7×10^−5^) for the additive model, that remained significant after adjusting for parity (p = 8×10^−5^). In addition to rs6434222 eight SNPs out of the seventeen examined in TFPI associated in both maternal and fetal samples. All of the associating SNPs in TFPI were also significant, using PRAT (Table 3). Only rs6434222 was significant after FDR correction.

**Figure 2:**
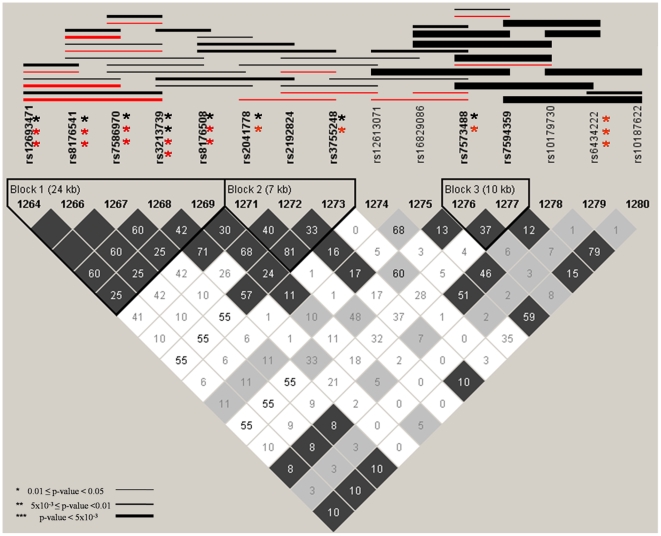
Maternal and fetal single locus and haplotype associations for TFPI. Asterisks to the right of a SNP indicates significant single locus allelic associations with PTB, red indicates significance in maternal samples and black indicates significance in fetal samples. The number of asterisks denotes the strength of significance. Lines denote a significant haplotype, red is for maternal samples and black is in fetal samples. The thickness of the line denotes the strength of significance. Only SNPs with MAF > 0.05 in maternal or fetal samples are presented in graph. Linkage disequilibrium plots (r^2^) are shown for fetal controls.

Several significant haplotype associations, mostly spanning two areas of the TFPI gene, were identified (Figure 2). One area includes five SNPs denoted by Block 1 (rs12693471, rs8176541, rs7586970, rs3213739 and rs8176508). All of these SNPs had suggestive associations in the single SNP analyses, and showed evidence of both maternal and fetal haplotype association. The other significant haplotype effects were in fetal samples and appear to be driven by rs6434222. Maternal single locus and haplotype associations were not observed for this SNP. In maternal and fetal controls there was weak linkage disequilibrium (LD) between the two regions of the genes defined by Block 1 and rs6434222, indicating two potentially independent effects within this gene (Figure S1).

### Corroboration of Results

**Figure 3:**
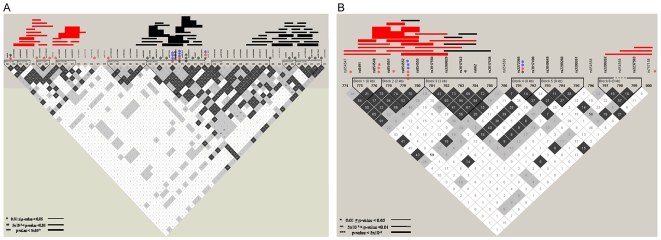
Significant single locus and haplotype associations in MoBa and Cenntennial studies. a) PTGER3 for maternal samples and b) PON1 for fetal samples. Asterisks to the right of a single nucleotide polymorphism indicates significant single locus allelic associations with preterm birth, blue denotes significance in pooled samples, red indicates significance in the MoBa study only and black indicates significance in the Cenn study only. All pooled results are significant after correction for multiple testing with FDR. The number of asterisks denotes the strength of significance. Lines denote a significant haplotype, red is for the MoBa study and black is for the Cenntennial study. The thickness of the line denotes the strength of significance. Linkage disequilibrium plots (r^2^) are shown for MoBa maternal controls for PTGER3 (a) and MoBa fetal controls for PON1 (b).

SNPs were included for combined analysis if the allele or genotype p-values were less than 0.05 in one study (MoBa or Cenn) and less than 0.20 in the other study. In maternal samples, 60 SNPs met the criterion for the combined analysis, 30 of which had significant allelic and/or genotypic p-values in the combined dataset after correction for multiple testing with FDR (Table S4). The most significant single locus result in maternal combined samples was in prostaglandin E receptor 3 (PTGER3) (Table 4). There were six SNPs in this gene that met the criteria for combined analysis and three of these (rs2072947, rs977214 and rs6665776) had significant allelic and/or genotypic results in the combined data, all of which were significant after correction with FDR; rs977214 also remained significant after Bonferroni correction. Two of these SNPs (rs977214 and rs6665776) strongly associated with PTB in the combined data (rs977214: genotypic p = 3×10^−4^; rs6665776: genotypic p = 5×10^−4^) (Table 4). The best model for rs977214 after correcting for study site and gravidity was the AG/GG genotypes relative to the AA genotype and resulted in an OR of 0.55 (95% CI = 0.37–0.82, p = 3×10^−3^), indicating a protective effect (Table 5). This SNP deviated from HWE in both cases and controls in the combined data set; however, the sign of the inbreeding coefficients (f) was negative in controls (−0.097) and positive in cases (0.130), indicating that the deviations from HWE were not due to genotyping error as cases and controls were randomly placed on each plate for genotyping, nor are these results due to mixing of population samples (Wahlund Effect). The best model for rs6665776 after correcting for study site and gravidity was CC genotype relative to the AA/AC genotypes and had an OR of 1.75 (95% CI = 1.18–2.60, p = 5×10^−3^) (Table 5). There was strong LD (r^2^≥0.95) between these two SNPs in both Cenn and MoBa maternal controls (Figures S2A and B). Haplotype associations spanned three general regions of the PTGER3 gene; two of these regions were significant only in the Cenn study and one of these regions was significant only in the Moba study (Figure 3A, Table S5); however, taken together they both support the association of this gene with PTB.

**Table 4:** Genetic associations in combined MoBa and Cenn samples.

Sample	Gene	SNP	Allele	Cenn Case Allele Freq	Cenn Cont Allele Freq	Cenn Allele p	Cenn Genotype p	MoBa Case Allele Freq	MoBa Cont Allele Freq	MoBa Allele p	MoBa Genotype p	Pooled Case Allele Freq	Pooled Cont Allele Freq	Pooled Allele p	Pooled Genotype p
Maternal	PTGER3	rs977214	G	0.09	0.13	0.17	5×10^−3^	0.06	0.09	0.06	0.04	0.07^1^	0.11^2^	0.02*	3×10^−4^ **
		rs6665776	A	0.09	0.12	0.24	0.01	0.06	0.09	0.06	0.04	0.07^1^	0.11	0.02*	5×10^−4^ *
		rs2072947	C	0.53	0.44	0.02	0.03	0.41	0.46	0.21	0.03	0.46	0.45	0.58*	0.04*
Fetal	PON1	rs854552	C	0.22	0.28	0.12	0.25	0.21	0.30	2×10^−3^	6×10^−3^	0.21	0.29	8×10^−4^ *	2×10^−3^ *
		rs2272365	G	0.19	0.13	0.05	0.05	0.17	0.12	0.04	0.12	0.18	0.13	5×10^−3^ *	0.01*

1 cases deviated from HWE at rs977214 (p = 0.02), rs6665776 (p = 0.02).

2 controls deviated from HWE at rs977214 (p = 0.05).

*Significant after correction for multiple testing with FDR (q = 0.2).

**Significant after correction for multiple testing with Bonferroni.

**Table 5:** Odds ratios for significant associations in pooled samples.

Sample	Gene	SNP	Genic Location	Model	OR	95% CI	Model p
Maternal	PTGER3	rs977214	Intron	AAvsAG/GG	0.55	0.37–0.82	3×10^−3^
		rs6665776	Intron	AA/ACvsCC	1.75	1.18–2.60	5×10^−3^
		rs2072947	Intron	CCvsCT/TT	0.68	0.47–0.97	0.03
Fetal	PON1	rs854552	3′UTR	CC/CTvsTT	1.32	1.13–1.53	4×10^−4^
		rs2272365	Intron	GG/GTvsTT	0.61	0.44–0.85	9×10^−4^

Logistic regression models adjusted for center of collection and gravidity. Center of collection was not significant for any of the models (p>0.1), gravidity was significant for all of the models (p<5×10^−3^).

In fetal samples, 44 SNPs met the criteria for the combined analysis, 28 of these SNPs had significant allelic and/or genotypic associations in the combined data after correction for multiple testing with FDR (Table S4). The most significant single locus association in pooled samples was in PON1 at rs854552 (allelic p = 8×10^−4^, genotypic p = 2×10^−3^). This SNP remained significant after correction for multiple testing with FDR. The best model after correcting for study site and gravidity compared the rs854552 TT genotype to the CC/CT genotypes and resulted in an OR of 1.32 (95% CI = 1.13–1.53, p = 4×10^−4^) (Table 5). The LD patterns were similar in Cenn and MoBa fetal controls (Figure S2C and D). There were very few haplotype or single locus associations in the Cenn study for PON1; however, there were strong haplotype associations in the MoBa data that is being driven by the single locus association at rs854552 (Figure 3B, Table S5).

## Discussion

Our study explored candidate genes in the four hypothesized preterm labor pathways [8] for associations with PTB and determined the effect size of associations that corroborated a second PTB cohort. In MoBa the only association significant after correction for multiple testing was in fetal samples at rs6434222 in TFPI. This SNP was not significantly associated with PTB in maternal samples and there was only weak LD in maternal controls with this SNP, whereas in fetal controls there was stronger LD between this SNP and other SNPs with significant associations (Figure S1). This indicates that this SNP may exhibit an independent fetal single locus association with PTB. This conclusion is supported by the strong fetal haplotype associations involving this SNP in fetal but not maternal samples. TFPI is involved in the complement coagulation pathway and inhibits Coagulation Factor 3 and Coagulation Factor 10, both of which are important for the production of Coagulation Factor 2 (F2), a critical component in clot formation. Overproduction of F2 can cause contractions and activate matrix metalloproteinases (MMPs) [22]–[24], possibly leading to degradation of the extracellular matrix (ECM). ECM degradation causes changes in the cervix and rupture of the membranes that may result in PTB. The single SNP associations may indicate TFPI as a novel candidate gene for PTB risk. While these associations did not corroborate in the Cenn population other genes in the complement coagulation pathway, particularly tPA were associated with PTB in previous reports, indicating that this pathway may be important in PTB [21].

Among the genes examined for association with PTB ∼19% (26 genes) had suggestive associations (p<0.05) in fetal but not in maternal samples. This may indicate a potential paternal genetic effect, at least in part, for some pathways involved in PTB. This is in contrast to recent studies, based on epidemiological data, indicating that paternal factors contribute little to the risk of PTB [25], [26]. However, to adequately understand the involvement of the maternal and paternal genome in PTB additional methods to examine either maternal and fetal samples simultaneously or trios consisting of paternal, maternal and fetal samples are needed. As better methods are developed to handle such analyses, particularly with respect to perinatal medicine, where both maternal and fetal genomes are involved in the disease outcome, this question can be explored in greater detail. While in general, the genetic contribution of paternal genes may be marginal, it is possible that particular pathways are influenced more by paternal genetic contributions.

**Figure 4:**
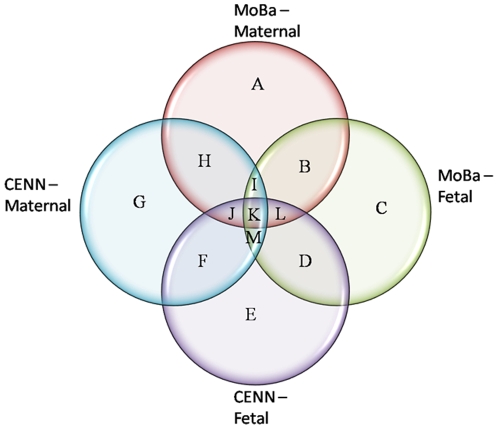
Identification of genes associated with PTB in multiple populations. Genetic associations are defined as any suggestive allelic or genotypic association (p<0.05) occurring in a given gene. These associations are uncorrected for multiple testing and are before correction for covariates such as parity. **A:** ADRB2, CARD15, CBS, CCL8, EPHX1, FAS, GSTP1, IL1B, IL1RN, IL2RB, IL8RA, MMP2, NFKBIE, PAFAH1B1, PGRMC2, PTGES **B:** ADH1B, MTHFR, NFKB1, PTGFR, SLC6A4, TLR4 **C:** CRH, F5, HSD11B1, HSD17B7, HSPA1A, IGFBP3, IL10RB, IL13, IL15, PGEA1, PGRMC1, PTGER2, SERPINE1, TCN2, TIMP4 **D:** HSPA6, IL1A, IL4, MMP8 **E:** CBS, GSTP1, HSPA14, IGF1, IL1B, IL1RN, IL2RB, NFKBIA, NFKBIE, NOS3, PAFAH1B1, PTGS2, SLC23A1 **F:** IL10RA, IL5, MMP1, MMP3 **G:** CCL2, CTLA4, DHFR, F5, IL10, PGRMC1, PTGS1, TLR2, TLR7, TNFRI, TNFRII, VEGF **H:** COL1A1, F7, IL18, IL1R2, IL6R, MTHFD1, NAT1, PLA2G4A **I:** COL5A1, IL1R1, IL4R **J:** KL, PLAT **K:** COL1A2, COL3A1, CYP19A1, IL1RAP, IL2RA, NFKBIB, PON2, PTGER3, TIMP3, TSHR, UGT1A1 **L:** CRHR2, EDN2, NR3C1, PGR, PON1, TREM1 **M:** COL5A2, CRHBP, EPHX2.

The genes that were suggestive for association with PTB in fetal (IL1A, IL4 and MMP8) and maternal samples (IL-1R2 and IL-6R) in both the MoBa and Cenn studies were mainly infection and inflammation genes (Figure 4, sector D). It is interesting to note that most associating fetal variants were in the cytokines genes themselves whereas maternal associations were generally in cytokine receptor genes (Figure 4, sector H). This suggests maternal polymorphisms may influence or modulate functional contributions of fetal SNPs in the inflammatory pathways. We speculate that fetal immunological response to the maternal environment may be affected by maternal variants, thereby regulating the impact of fetal encoded risks through complex interactions. Further studies, particularly genome-wide association studies and inclusion of paternal samples will be essential for elucidating the relative contributions of paternal and fetal genotypes to the risk of PTB.

In our study several genes from multiple biological pathways had modest to strong associations with PTB, and many of these corroborated an independent data set on PTB. While this study did not directly test replication, results from the combined analyses indicate genes with universal or generalized associations. The most significant associations in the combined samples were from two major PTB pathways: 1) prostaglandin (PG) effectors (PTGER3) in maternal samples that influence PG mediated uterotonic activity and 2) decidual hemorrhage (PON1) in fetal samples. PTGER3 is a receptor required for prostaglandin response that plays a role in initiation of contraction (labor). Activation of the inflammatory process has been documented to increase prostaglandins[27], [28]. Several SNPs in PTGER3 were significant in the combined MoBa and Cenn maternal samples, supporting the role of variation in this contractile agent in the final effecter pathway and PTB. However, we note that the patterns of haplotype association in MoBa and Cenn differed. Although there is some evidence for differences in LD pattern between these two cohorts, we do not think that the differences are enough to motivate such strong differences in haplotype associations, especially since there is some evidence for SNPs associating in both studies in the middle portion of the gene. Rather it is possible that our data support allelic heterogeneity across the two studies.

The second pathway that associated with PTB in the two cohorts is the decidual hemorrhage pathway that can potentially activate biochemical mediators such as matrix metalloproteinases and cause ECM degradation. PON1 is a member of the paraoxonase gene family and it can impact preterm labor in multiple ways. Primarily, mutations in this gene are associated with changes in lipid profiles (high-density lipoprotein (HDL) low-density lipoprotein (LDL)) [29], [30]. HDL increases the production of prostaglandin E_2_, leading to contractions [31]. Additionally, hypothesized mutations in this gene could indirectly disrupt thrombotic pathways and activation of thrombin and plasminogen cascade can activate MMPs and MMP mediated ECM degradation [31]–[33]. A previous study examining one mutation in PON1 (rs662) found an association in fetal but not maternal Chinese samples [31]. While this SNP was not associated in the Cenn, MoBa or pooled samples we did identify a different SNP (rs854552) located in the 3'UTR of the gene that was strongly associated with PTB in the pooled fetal samples. It is difficult to conclude that this SNP replicates the association found in the Chinese samples; however, this gene does replicate in two populations of European descent and the Chinese population, indicating perhaps allelic heterogeneity for PON1, but more importantly a consistent role for PON1 in PTB.

Our studies were limited in that they are candidate gene studies; therefore many associations may be missed, including genes representing novel biology with respect to PTB. In addition, the case-control criteria for the MoBa and Cenn studies were slightly different and while there were still many associations discovered, studies with more carefully defined phenotypic criteria are needed to validate these results. Finally, we emphasize that although some results were found to be consistent across MoBa and Cenn, there was evidence for different patterns of association. This was especially pronounced for TFPI, which did not corroborate, and PTGER3 haplotype analyses that showed differences in genic region that associated with PTB. This may be due to underlying population differences between our samples, possibly allele frequency differences that impact power. Alternatively, there could be population specific risk alleles that were indirectly detected through our analyses. Lastly, it is possible that the underlying genetic models for PTB are multi-factorial and that the ability to detect single locus association is impacted by changes in a second or third factor that were not corrected measured or corrected for in our analyses [34].

Despite its potential limitations this study identified multiple genes with significant or marginally significant associations with PTB in two separate samples. When samples were combined, multiple SNPs with strong allelic or genotypic p-values were identified, including SNPs involved in inflammation and infection, ECM degradation and decidual hemorrhage pathways. This study validated a previously identified genetic association with PTB and further studies can address the biological role these genes play.

## Materials and Methods

### Subjects

The Norwegian Mother and Child Cohort (MoBa) is a national pregnancy cohort designed to explore the causes of complex pregnancy related phenotypes. The main objective of this cohort is to develop a long term resource to test hypotheses relating to pregnancy outcomes and childhood health. More than 100,000 pregnancies have been included during the study period of 1999–2008 by the Norwegian Institute of Public Health. The target population consisted of all pregnant women in Norway. Women were invited to participate during their routine ultrasound scan at gestational week 17–18. All hospitals and maternity units with more than 100 deliveries annually were invited to participate, and 50 of 52 such units participated. The total participation rate was 42.7% [35]. Epidemiological data and biological material have been and will continue to be collected from both the mothers and the children. The MoBa study collected data on nutrition, overall health status, and child outcome. In addition to these variables additional data were added from the Medical Birth Registry of Norway. The MoBa study has been described in detail elsewhere [35]. The present study used samples derived from a subset of the MoBa cohort (version 2) available at its outset (53,711 pregnancies) [36].

Data from the Medical Birth Registry on gestational age, type of delivery, intrauterine fetal death, congenital malformations and plurality were used. Gestational age was based on ultrasound in early second trimester (week 16–19) and/or the date of last menstrual period. However, ultrasound was the main method of determining gestational age as most pregnancies in Norway are dated by ultrasound.

The cases in this study were defined as singletons with spontaneous onset of PTB between 22 gestational weeks and 0 out of 7 days (22^0/7^) and 36^6/7^ gestational weeks in women aged 20–35 years. This age range was chosen to ensure that women at high risk for PTB because of age (young women less than 20 years of age and older women greater than 35 years of age) were not included in the study. Type of delivery was recorded as spontaneous labor, induced labor, or pre-labor caesarean section. Women with induced labor and pre-labor caesarean section groups, intrauterine fetal death, plurality and if the fetus had congenital malformations were all excluded. The definitions of these conditions have been previously described [7]. Other exclusion criteria were pre-existing medical conditions such as diabetes, hypertension, autoimmune diseases, inflammatory bowel diseases, systemic lupus erythematosus, rheumatoid arthritis, scleroderma or any immune-compromised condition. Women were also excluded if they had pregnancy complications such as preeclampsia, hypertension, diabetes, small for gestational age (according to intrauterine growth curves), abruption of the placenta, placenta previa, cervical cerclage or fetal malformations [37]. The controls were selected according to the same criteria as above except for spontaneous onset of term birth with gestational age between 39^0/7^ and 40^6/7^ weeks. The Regional Committee for Medical Research and Ethics (S-06075) approved the MoBa study and approval was also achieved from the Norwegian Data Inspectorate.

For this study 434 mother-baby pairs (214 cases and 220 controls) were selected for analysis from MoBa. A previously published study on PTB was used to corroborate results from our study. This study utilized samples from the Centennial study (Cenn) collected in Nashville, TN and is described in detail elsewhere [21]. This sample consisted of maternal and fetal individuals of both European (European American – EA) and African (African-American) descent, but only the EA samples were used for corroboration. The combined datasets contained 764 maternal samples (353 cases and 411 controls) and 738 fetal samples (343 cases and 395 controls).

### DNA Genotyping and Quality Control

A total of 1,536 tag SNPs were selected from 143 PTB candidate genes. Of these 1,404 SNPs from 129 genes were chosen because they had been included in a previous study of PTB [21]. In addition, 132 SNPs from 14 genes in the complement-coagulation pathway were added to the present study because this pathway was associated with PTB previously. SNPs in Velez et al[21] were selected based on their ability to tag surrounding variants in the Caucasian and Yoruban populations of the HapMap database (http://www.hapmap.org). A minor allele frequency of 0.07 in CEPH and 0.20 in YRI and an r^2^ of 0.80 was used to determine tagSNPs. The additional SNPs were selected as tagSNPs in the CEPH, based on similar criteria. Genotyping was performed on the Illumina GoldenGate Assay system. 1,430 SNPs encompassing 140 candidate genes were successfully genotyped with genotyping efficiency greater than 95% (Table S1).

Both maternal and fetal samples were excluded if the last menstrual period and birth weight were not consistent with case-control status, suggestive of sample misidentification or if extracted DNA was not available in the Biobank. Additionally, samples were excluded if greater than 1% of the SNPs had Mendelian errors. This was done to ensure high quality genotyping accuracy. Additionally, individuals were excluded for low genotyping efficiency (<95%). The final sample size included 419 fetal samples (203 cases, 216 controls) and 424 maternal samples (207 cases, 217 controls).

### DNA Genotyping: Corroboration of Results

A total of 1,316 SNPs in the present study overlapped with the previous study on PTB in EA maternal and fetal samples (Table S1). Additionally, 38 SNPs from three genes (plasminogen [PLG], methionine synthase reductase [MTRR] and tissue factor pathway inhibitor [TFPI]) were successfully genotyped (genotyping efficiency >95%) in the Cenn samples, using the Sequenom platform. A total of 304 maternal and 272 fetal samples were successfully genotyped on this platform with >85% genotyping efficiency. These genes were not originally included in the Cenn analysis; however, significant associations were identified in the MoBa samples and the Cenn samples were reanalyzed for association with these SNPs to determine if these associations could be replicated (Table S2). Institutional Review Boards at TriStar Nashville, TN and Vanderbilt University, Nashville, TN approved this study.

### Statistical Analysis: MoBa

Chi-square tests were used to examine differences between cases and controls for APGAR at 1 minute (<7 versus ≥7), APGAR score at 5 minutes (<7 versus ≥7) and smoking (non-smokers versus smokers). Parity (number of times a woman has given birth defined by birth registry), gestational age (days), birth weight (grams) and maternal age were tested for normality using Shapiro-Wilks tests. None of these variables were normally distributed; therefore, these variables were analyzed with nonparametric Mann-Whitney two-sample ranksum tests. Stata version 10 statistical software was used for all analyses (StataCorp, College Station, TX).

Gene names, marker positions (base pair – bp) and marker function were identified using the SNPper database (http://snpper.chip.org). Allele and genotype differences between cases and controls, deviations in Hardy-Weinberg equilibrium (HWE) and inbreeding coefficients (f) were calculated with PLINK (http://pngu.mgh.harvard.edu/purcell/plink/)[38], the Whole-genome Association Study Pipeline (WASP) developed at the Center for Human Genetics Research of Vanderbilt University (http://chgr.mc.vanderbilt.edu/wasp/) and Powermarker software programs [39]. Statistical significance for these analyses was determined using chi-square tests or Fisher's exact tests (if there were less than five individuals for any allele or genotype). These results were corroborated using a prevalence based association test (PRAT) [40]. This test calculates a global allele frequency based on the prevalence of the disease in order to calculate expected frequencies of each genotype in cases and controls separately, and a permutation test is used to determine the significance. The global allele frequency was determined using a PTB prevalence of 5% in the Norwegian cohort. False discovery rate (q = 0.2) was used to correct for multiple testing in maternal and fetal samples, separately [41].

The most significant allelic and/or genotypic associations were followed up with additive, dominant and recessive genotype models, using the minor allele as the risk allele, using logistic regression. Significant (p<0.05) logistic regression models were adjusted for parity because this was the only risk factor that was significantly different between cases and controls in MoBa. These analyses were performed with Stata version 10. Graphical representation of significant results and haplotypes was performed with Haploview [42].

### Statistical Analysis: Corroboration of Results

The MoBa and Cenn samples were combined for SNPs that met the criteria described below, and analyzed for allelic and genotypic association. SNPs were included for combined analysis if the allele or genotype p-values were less than 0.05 in one study (MoBa or Cenn) and less than 0.20 in the other study. This criterion, while arbitrary, was designed to capture associations that may have been missed due to inadequate power in an individual study. However, this criterion was also designed to exclude associations that were primarily driven by only one of the two studies. Genotype and allele associations were corrected for multiple testing in maternal and fetal sample separately with FDR (q = 0.2). An effect size was determined with logistic regression for additive, recessive and dominant models again, using the minor allele as the risk allele and correcting for site of collection and gravidity.

The genes with the most significant single SNP associations in maternal and fetal samples were followed-up with 2, 3 and 4 marker sliding window haplotype analysis using Unphased [43]. This software uses a quasi-Newton algorithm to maximize the likelihood of each haplotype when an individual's phase is unknown. The overall test of association is a likelihood ratio test based on the null hypothesis that all of the haplotypes have equal odds ratios. Haplotype analysis was performed in the MoBa and Cenn samples separately.

## Supporting Information

Figure S1 Linkage disequilibrium plots (r2) are shown for maternal (a) and fetal (b) controls for TFPI.
(0.48 MB TIF)

Figure S2 Linkage disequilibrium plots (r2) are shown for a) Cenn maternal PTGER3 b) Moba maternal PTGER3 c) Cenn fetal PON1 d) Moba fetal PON1.
(1.00 MB TIF)

Table S1 (0.83 MB DOC)

Table S2 (0.13 MB DOC)

Table S3 (0.32 MB DOC)

Table S4 (0.39 MB DOC)

Table S5 (0.20 MB RTF)
