# Supplementary figures and images for: Maternal and Fetal Genetic Associations of PTGER3 and PON1 with Preterm Birth
Source: PLoS One. 2010 Feb 3;5(2):e9040. doi: 10.1371/journal.pone.0009040 (PMC2815792; doi:10.1371/journal.pone.0009040)

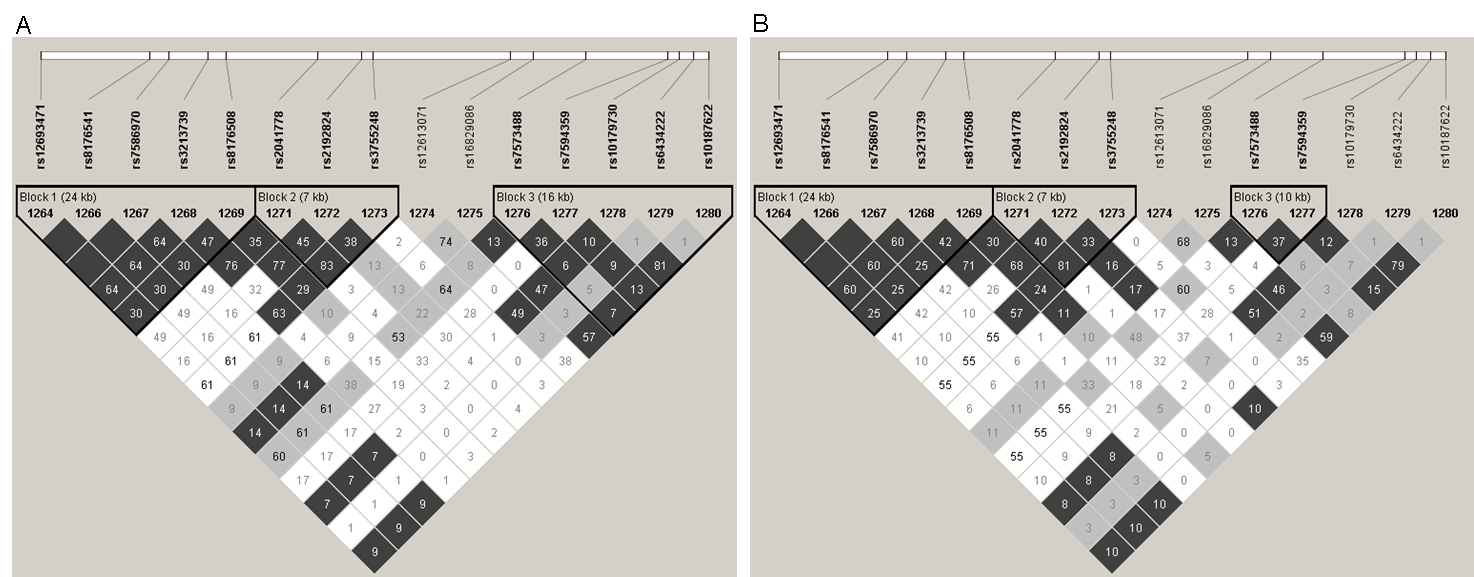

Supplement: Figure S1 — Linkage disequilibrium plots (r2) are shown for maternal (a) and fetal (b) controls for TFPI. (0.48 MB TIF) [file pone.0009040.s001.tif]

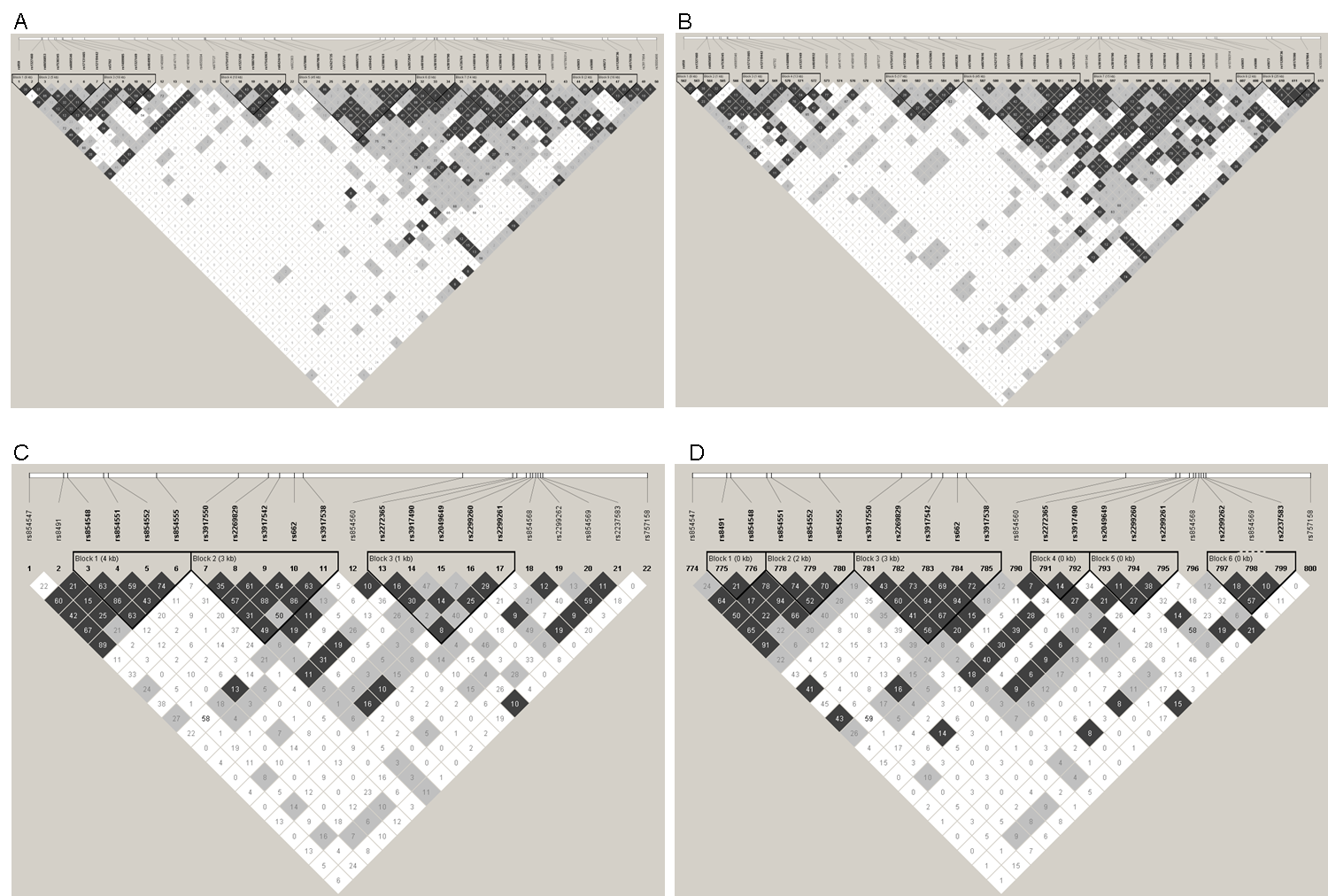

Supplement: Figure S2 — Linkage disequilibrium plots (r2) are shown for a) Cenn maternal PTGER3 b) Moba maternal PTGER3 c) Cenn fetal PON1 d) Moba fetal PON1. (1.00 MB TIF) [file pone.0009040.s002.tif]
