# Supplementary material for: Maternal and Fetal Genetic Associations of PTGER3 and PON1 with Preterm Birth
Source: PLoS One. 2010 Feb 3;5(2):e9040. doi: 10.1371/journal.pone.0009040 (PMC2815792; doi:10.1371/journal.pone.0009040)
Supplement: Table S1 — (0.83 MB DOC) [file pone.0009040.s003.doc]

Supplemental Table 1: TagSNPs analyzed in MoBa cohort.

| RS# | Gene | RS# | Gene | RS# | Gene |
| --- | --- | --- | --- | --- | --- |
| **rs12451328** | **ACE** | **rs1143630** | **IL1B** | **rs3733260** | **PGRMC2** |
| **rs4267385** | **ACE** | **rs1143634** | **IL1B** | **rs4975180** | **PGRMC2** |
| **rs4295** | **ACE** | **rs1143643** | **IL1B** | **rs4975220** | **PGRMC2** |
| **rs4305** | **ACE** | **rs2853550** | **IL1B** | **rs10157410** | **PLA2G4A** |
| **rs4311** | **ACE** | **rs4848306** | **IL1B** | **rs10911946** | **PLA2G4A** |
| **rs4362** | **ACE** | **rs2110726** | **IL1R1** | **rs11587539** | **PLA2G4A** |
| **rs4459610** | **ACE** | **rs2160227** | **IL1R1** | **rs12404877** | **PLA2G4A** |
| **rs4461142** | **ACE** | **rs2287047** | **IL1R1** | **rs12720497** | **PLA2G4A** |
| **rs1042026** | **ADH1B** | **rs3171845** | **IL1R1** | **rs12726519** | **PLA2G4A** |
| **rs1159918** | **ADH1B** | **rs3732131** | **IL1R1** | **rs12749354** | **PLA2G4A** |
| **rs1229982** | **ADH1B** | **rs3771202** | **IL1R1** | **rs1569479** | **PLA2G4A** |
| **rs12507573** | **ADH1B** | **rs3917225** | **IL1R1** | **rs17591814** | **PLA2G4A** |
| **rs13133908** | **ADH1B** | **rs3917254** | **IL1R1** | **rs1980444** | **PLA2G4A** |
| **rs1353621** | **ADH1B** | **rs3917273** | **IL1R1** | **rs2049963** | **PLA2G4A** |
| **rs1693457** | **ADH1B** | **rs3917292** | **IL1R1** | **rs2076075** | **PLA2G4A** |
| **rs17033** | **ADH1B** | **rs3917296** | **IL1R1** | **rs2223307** | **PLA2G4A** |
| **rs1789882** | **ADH1B** | **rs3917304** | **IL1R1** | **rs2223309** | **PLA2G4A** |
| **rs4147536** | **ADH1B** | **rs3917306** | **IL1R1** | **rs4402086** | **PLA2G4A** |
| **rs1229980** | **ADH1C** | **rs3917332** | **IL1R1** | **rs4651330** | **PLA2G4A** |
| **rs1614972** | **ADH1C** | **rs949963** | **IL1R1** | **rs6656909** | **PLA2G4A** |
| **rs17586163** | **ADH1C** | **rs951193** | **IL1R1** | **rs6683416** | **PLA2G4A** |
| **rs3762896** | **ADH1C** | **rs1108338** | **IL1R2** | **rs6683515** | **PLA2G4A** |
| **rs904096** | **ADH1C** | **rs11884283** | **IL1R2** | **rs6685652** | **PLA2G4A** |
| **rs1042713** | **ADRB2** | **rs12467316** | **IL1R2** | **rs6695515** | **PLA2G4A** |
| **rs12654778** | **ADRB2** | **rs2072474** | **IL1R2** | **rs6696406** | **PLA2G4A** |
| **rs1432622** | **ADRB2** | **rs2160140** | **IL1R2** | **rs726706** | **PLA2G4A** |
| **rs4705271** | **ADRB2** | **rs2302589** | **IL1R2** | **rs7526089** | **PLA2G4A** |
| **rs17312836** | **CARD15** | **rs3218861** | **IL1R2** | **rs7540602** | **PLA2G4A** |
| **rs2066843** | **CARD15** | **rs3218883** | **IL1R2** | **rs7545121** | **PLA2G4A** |
| **rs4785224** | **CARD15** | **rs3218927** | **IL1R2** | **rs7555140** | **PLA2G4A** |
| **rs5743289** | **CARD15** | **rs3218979** | **IL1R2** | **rs761517** | **PLA2G4A** |
| **rs5743291** | **CARD15** | **rs4141134** | **IL1R2** | **rs932476** | **PLA2G4A** |
| **rs7203344** | **CARD15** | **rs4321386** | **IL1R2** | **rs2020919** | **PLAT** |
| **rs751271** | **CARD15** | **rs4851520** | **IL1R2** | **rs2020922** | **PLAT** |
| **rs8056611** | **CARD15** | **rs4851522** | **IL1R2** | **rs2299609** | **PLAT** |
| **rs1051319** | **CBS** | **rs4851526** | **IL1R2** | **rs4471024** | **PLAT** |
| **rs11203172** | **CBS** | **rs4851527** | **IL1R2** | **rs4581040** | **PLAT** |
| **rs11701048** | **CBS** | **rs4851531** | **IL1R2** | **rs7837156** | **PLAT** |
| **rs12329764** | **CBS** | **rs6543105** | **IL1R2** | **rs879293** | **PLAT** |
| **rs1788484** | **CBS** | **rs733498** | **IL1R2** | rs2227551 | PLAU |
| **rs234705** | **CBS** | **rs7561191** | **IL1R2** | rs2227562 | PLAU |
| **rs234715** | **CBS** | **rs7589525** | **IL1R2** | rs2227564 | PLAU |
| **rs3788050** | **CBS** | **rs1015704** | **IL1RAP** | rs2461863 | PLAU |
| **rs6586282** | **CBS** | **rs1015705** | **IL1RAP** | rs34930250 | PLAU |
| **rs706208** | **CBS** | **rs1024941** | **IL1RAP** | rs3805118 | PLAU |
| **rs1024610** | **CCL2** | **rs1024946** | **IL1RAP** | rs2239372 | PLAUR |
| **rs3760396** | **CCL2** | **rs1024949** | **IL1RAP** | rs2283628 | PLAUR |
| **rs4586** | **CCL2** | **rs1035347** | **IL1RAP** | rs2286960 | PLAUR |
| **rs991804** | **CCL2** | **rs10513854** | **IL1RAP** | rs2302524 | PLAUR |
| **rs1634491** | **CCL3** | **rs10937439** | **IL1RAP** | rs344779 | PLAUR |
| **rs1634502** | **CCL3** | **rs10937442** | **IL1RAP** | rs344781 | PLAUR |
| **rs1851503** | **CCL3** | **rs11915384** | **IL1RAP** | rs344787 | PLAUR |
| **rs9972960** | **CCL3** | **rs11929157** | **IL1RAP** | rs397374 | PLAUR |
| **rs11575060** | **CCL8** | **rs12053868** | **IL1RAP** | rs4251831 | PLAUR |
| **rs1233650** | **CCL8** | **rs1469007** | **IL1RAP** | rs4251854 | PLAUR |
| **rs3138039** | **CCL8** | **rs16865597** | **IL1RAP** | rs4251864 | PLAUR |
| **rs4794999** | **CCL8** | **rs1988743** | **IL1RAP** | rs4251938 | PLAUR |
| **rs885691** | **CCL8** | **rs2059020** | **IL1RAP** | rs4802189 | PLAUR |
| **rs2569190** | **CD14** | **rs2193880** | **IL1RAP** | rs4803648 | PLAUR |
| **rs2569193** | **CD14** | **rs2241343** | **IL1RAP** | rs11060 | PLG |
| **rs1061237** | **COL1A1** | **rs2885370** | **IL1RAP** | rs1950562 | PLG |
| **rs1107946** | **COL1A1** | **rs3773953** | **IL1RAP** | rs2314852 | PLG |
| **rs2075559** | **COL1A1** | **rs3773958** | **IL1RAP** | rs4252092 | PLG |
| **rs2269336** | **COL1A1** | **rs3773976** | **IL1RAP** | rs4252125 | PLG |
| **rs2277632** | **COL1A1** | **rs3773977** | **IL1RAP** | rs4252159 | PLG |
| **rs2586482** | **COL1A1** | **rs3773981** | **IL1RAP** | rs4252166 | PLG |
| **rs2586485** | **COL1A1** | **rs3773982** | **IL1RAP** | rs783144 | PLG |
| **rs2586488** | **COL1A1** | **rs3773983** | **IL1RAP** | rs783147 | PLG |
| **rs2696247** | **COL1A1** | **rs3773989** | **IL1RAP** | rs783166 | PLG |
| **rs2857396** | **COL1A1** | **rs3773990** | **IL1RAP** | rs783176 | PLG |
| **rs10046552** | **COL1A2** | **rs3773994** | **IL1RAP** | rs813641 | PLG |
| **rs1062394** | **COL1A2** | **rs3821744** | **IL1RAP** | rs9295131 | PLG |
| **rs11764718** | **COL1A2** | **rs4140711** | **IL1RAP** | rs9458011 | PLG |
| **rs11765563** | **COL1A2** | **rs4320092** | **IL1RAP** | rs9458023 | PLG |
| **rs11982782** | **COL1A2** | **rs4624606** | **IL1RAP** | **rs6713532** | **POMC** |
| **rs12668754** | **COL1A2** | **rs4686554** | **IL1RAP** | **rs17166818** | **PON1** |
| **rs13234022** | **COL1A2** | **rs4687151** | **IL1RAP** | **rs2049649** | **PON1** |
| **rs17166249** | **COL1A2** | **rs4687154** | **IL1RAP** | **rs2237583** | **PON1** |
| **rs1800222** | **COL1A2** | **rs4687163** | **IL1RAP** | **rs2269829** | **PON1** |
| **rs2472** | **COL1A2** | **rs6444435** | **IL1RAP** | **rs2272365** | **PON1** |
| **rs2521205** | **COL1A2** | **rs6765375** | **IL1RAP** | **rs2299260** | **PON1** |
| **rs2621213** | **COL1A2** | **rs6781037** | **IL1RAP** | **rs2299261** | **PON1** |
| **rs369982** | **COL1A2** | **rs759783** | **IL1RAP** | **rs2299262** | **PON1** |
| **rs3736638** | **COL1A2** | **rs7615368** | **IL1RAP** | **rs3735590** | **PON1** |
| **rs3763466** | **COL1A2** | **rs7615533** | **IL1RAP** | **rs3917490** | **PON1** |
| **rs3814967** | **COL1A2** | **rs7626071** | **IL1RAP** | **rs3917538** | **PON1** |
| **rs388625** | **COL1A2** | **rs7626795** | **IL1RAP** | **rs3917542** | **PON1** |
| **rs389328** | **COL1A2** | **rs7628250** | **IL1RAP** | **rs3917550** | **PON1** |
| **rs400218** | **COL1A2** | **rs7628333** | **IL1RAP** | **rs662** | **PON1** |
| **rs406226** | **COL1A2** | **rs7650510** | **IL1RAP** | **rs757158** | **PON1** |
| **rs411717** | **COL1A2** | **rs9290936** | **IL1RAP** | **rs8491** | **PON1** |
| **rs420257** | **COL1A2** | **rs9290939** | **IL1RAP** | **rs854547** | **PON1** |
| **rs42521** | **COL1A2** | **rs929729** | **IL1RAP** | **rs854548** | **PON1** |
| **rs42523** | **COL1A2** | **rs9817203** | **IL1RAP** | **rs854551** | **PON1** |
| **rs42524** | **COL1A2** | **rs9821122** | **IL1RAP** | **rs854552** | **PON1** |
| **rs42527** | **COL1A2** | **rs9831803** | **IL1RAP** | **rs854555** | **PON1** |
| **rs42528** | **COL1A2** | **rs9845825** | **IL1RAP** | **rs854560** | **PON1** |
| **rs42531** | **COL1A2** | **rs9847868** | **IL1RAP** | **rs854568** | **PON1** |
| **rs4266** | **COL1A2** | **rs9849030** | **IL1RAP** | **rs854569** | **PON1** |
| **rs441051** | **COL1A2** | **rs9875362** | **IL1RAP** | **rs11977702** | **PON2** |
| **rs6465412** | **COL1A2** | **rs9877268** | **IL1RAP** | **rs11981433** | **PON2** |
| **rs760043** | **COL1A2** | **rs9883249** | **IL1RAP** | **rs2237585** | **PON2** |
| **rs7781954** | **COL1A2** | **rs17042917** | **IL1RN** | **rs2286232** | **PON2** |
| **rs7804898** | **COL1A2** | **rs1794066** | **IL1RN** | **rs2286233** | **PON2** |
| **rs10204508** | **COL3A1** | **rs2637988** | **IL1RN** | **rs2299266** | **PON2** |
| **rs12693525** | **COL3A1** | **rs315920** | **IL1RN** | **rs2299267** | **PON2** |
| **rs13306267** | **COL3A1** | **rs315942** | **IL1RN** | **rs43037** | **PON2** |
| **rs1516454** | **COL3A1** | **rs315943** | **IL1RN** | **rs6978425** | **PON2** |
| **rs16830973** | **COL3A1** | **rs315946** | **IL1RN** | **rs730365** | **PON2** |
| **rs17358825** | **COL3A1** | **rs315949** | **IL1RN** | **rs7802018** | **PON2** |
| **rs1878201** | **COL3A1** | **rs315951** | **IL1RN** | **rs9641164** | **PON2** |
| **rs1914037** | **COL3A1** | **rs3213448** | **IL1RN** | **rs987539** | **PON2** |
| **rs2138533** | **COL3A1** | **rs380092** | **IL1RN** | **rs16896153** | **PTCRA** |
| **rs2203602** | **COL3A1** | **rs4251961** | **IL1RN** | **rs2234185** | **PTCRA** |
| **rs2271682** | **COL3A1** | **rs579543** | **IL1RN** | **rs6901007** | **PTCRA** |
| **rs3134646** | **COL3A1** | **rs928940** | **IL1RN** | **rs9471960** | **PTCRA** |
| **rs3134656** | **COL3A1** | **rs10027390** | **IL2** | **rs12147805** | **PTGER2** |
| **rs3736487** | **COL3A1** | **rs2069762** | **IL2** | **rs1254593** | **PTGER2** |
| **rs4667256** | **COL3A1** | **rs2069771** | **IL2** | **rs1254600** | **PTGER2** |
| **rs4667258** | **COL3A1** | **rs2069772** | **IL2** | **rs1390376** | **PTGER2** |
| **rs7576108** | **COL3A1** | **rs2069778** | **IL2** | **rs708498** | **PTGER2** |
| **rs7579903** | **COL3A1** | **rs2069779** | **IL2** | **rs708505** | **PTGER2** |
| **rs10114036** | **COL5A1** | **rs4833248** | **IL2** | **rs708506** | **PTGER2** |
| **rs10745387** | **COL5A1** | **rs10795737** | **IL2RA** | **rs10789314** | **PTGER3** |
| **rs10776908** | **COL5A1** | **rs1107345** | **IL2RA** | **rs11209736** | **PTGER3** |
| **rs11103509** | **COL5A1** | **rs11256457** | **IL2RA** | **rs12119442** | **PTGER3** |
| **rs11103543** | **COL5A1** | **rs11256497** | **IL2RA** | **rs1327449** | **PTGER3** |
| **rs11999194** | **COL5A1** | **rs11598648** | **IL2RA** | **rs1327460** | **PTGER3** |
| **rs12002679** | **COL5A1** | **rs12244380** | **IL2RA** | **rs1327466** | **PTGER3** |
| **rs12005720** | **COL5A1** | **rs12359875** | **IL2RA** | **rs1409164** | **PTGER3** |
| **rs13946** | **COL5A1** | **rs12722486** | **IL2RA** | **rs1409165** | **PTGER3** |
| **rs3124291** | **COL5A1** | **rs12722563** | **IL2RA** | **rs1409981** | **PTGER3** |
| **rs3124932** | **COL5A1** | **rs12722588** | **IL2RA** | **rs1409985** | **PTGER3** |
| **rs3128597** | **COL5A1** | **rs12722596** | **IL2RA** | **rs17131465** | **PTGER3** |
| **rs3128621** | **COL5A1** | **rs12722605** | **IL2RA** | **rs17131487** | **PTGER3** |
| **rs3811151** | **COL5A1** | **rs2031229** | **IL2RA** | **rs17541722** | **PTGER3** |
| **rs3811152** | **COL5A1** | **rs2076846** | **IL2RA** | **rs17542063** | **PTGER3** |
| **rs3811153** | **COL5A1** | **rs2228150** | **IL2RA** | **rs1887404** | **PTGER3** |
| **rs3811161** | **COL5A1** | **rs2386841** | **IL2RA** | **rs2050066** | **PTGER3** |
| **rs4841937** | **COL5A1** | **rs2476491** | **IL2RA** | **rs2072947** | **PTGER3** |
| **rs4842151** | **COL5A1** | **rs3134883** | **IL2RA** | **rs2256385** | **PTGER3** |
| **rs4842161** | **COL5A1** | **rs4749926** | **IL2RA** | **rs2300161** | **PTGER3** |
| **rs4842167** | **COL5A1** | **rs6602392** | **IL2RA** | **rs2300164** | **PTGER3** |
| **rs4842172** | **COL5A1** | **rs6602398** | **IL2RA** | **rs2300167** | **PTGER3** |
| **rs10165260** | **COL5A2** | **rs706778** | **IL2RA** | **rs2421735** | **PTGER3** |
| **rs10191420** | **COL5A2** | **rs706779** | **IL2RA** | **rs2817864** | **PTGER3** |
| **rs10497699** | **COL5A2** | **rs7072398** | **IL2RA** | **rs3000466** | **PTGER3** |
| **rs11691604** | **COL5A2** | **rs7072793** | **IL2RA** | **rs3819783** | **PTGER3** |
| **rs12611950** | **COL5A2** | **rs7073236** | **IL2RA** | **rs3819790** | **PTGER3** |
| **rs13024858** | **COL5A2** | **rs7093069** | **IL2RA** | **rs4147115** | **PTGER3** |
| **rs1515864** | **COL5A2** | **rs7910961** | **IL2RA** | **rs4649932** | **PTGER3** |
| **rs3923384** | **COL5A2** | **rs791590** | **IL2RA** | **rs4650094** | **PTGER3** |
| **rs6434317** | **COL5A2** | **rs942200** | **IL2RA** | **rs481940** | **PTGER3** |
| **rs6434322** | **COL5A2** | **rs9663421** | **IL2RA** | **rs5673** | **PTGER3** |
| **rs6752781** | **COL5A2** | **rs1003694** | **IL2RB** | **rs5680** | **PTGER3** |
| **rs6760780** | **COL5A2** | **rs2072861** | **IL2RB** | **rs5693** | **PTGER3** |
| **rs7420331** | **COL5A2** | **rs2235330** | **IL2RB** | **rs5697** | **PTGER3** |
| **rs9288163** | **COL5A2** | **rs2281094** | **IL2RB** | **rs5702** | **PTGER3** |
| **rs10098823** | **CRH** | **rs228937** | **IL2RB** | **rs578096** | **PTGER3** |
| **rs10105164** | **CRH** | **rs228945** | **IL2RB** | **rs594454** | **PTGER3** |
| **rs3176921** | **CRH** | **rs228947** | **IL2RB** | **rs602383** | **PTGER3** |
| **rs6472257** | **CRH** | **rs228954** | **IL2RB** | **rs6424410** | **PTGER3** |
| **rs6996265** | **CRH** | **rs228957** | **IL2RB** | **rs6424414** | **PTGER3** |
| **rs7839698** | **CRH** | **rs228968** | **IL2RB** | **rs6656853** | **PTGER3** |
| **rs10055255** | **CRHBP** | **rs228975** | **IL2RB** | **rs6665776** | **PTGER3** |
| **rs10514082** | **CRHBP** | **rs2743827** | **IL2RB** | **rs6670616** | **PTGER3** |
| **rs1875999** | **CRHBP** | **rs3218264** | **IL2RB** | **rs6678886** | **PTGER3** |
| **rs32897** | **CRHBP** | **rs3218292** | **IL2RB** | **rs6685546** | **PTGER3** |
| **rs6453267** | **CRHBP** | **rs3218295** | **IL2RB** | **rs726764** | **PTGER3** |
| **rs17689966** | **CRHR1** | **rs3218312** | **IL2RB** | **rs7530345** | **PTGER3** |
| **rs878887** | **CRHR1** | **rs3218315** | **IL2RB** | **rs7541092** | **PTGER3** |
| **rs12701020** | **CRHR2** | **rs3218329** | **IL2RB** | **rs8179390** | **PTGER3** |
| **rs2190242** | **CRHR2** | **rs3218339** | **IL2RB** | **rs875727** | **PTGER3** |
| **rs2240404** | **CRHR2** | **rs84460** | **IL2RB** | **rs959** | **PTGER3** |
| **rs2267716** | **CRHR2** | **rs2070874** | **IL4** | **rs977214** | **PTGER3** |
| **rs2267717** | **CRHR2** | **rs2227284** | **IL4** | **rs10739757** | **PTGES** |
| **rs2284217** | **CRHR2** | **rs2243248** | **IL4** | **rs10988496** | **PTGES** |
| **rs2284219** | **CRHR2** | **rs2243263** | **IL4** | **rs11999368** | **PTGES** |
| **rs4722999** | **CRHR2** | **rs2243268** | **IL4** | **rs2241270** | **PTGES** |
| **rs6462219** | **CRHR2** | **rs2243274** | **IL4** | **rs2302821** | **PTGES** |
| **rs929377** | **CRHR2** | **rs2243290** | **IL4** | **rs4636306** | **PTGES** |
| **rs973002** | **CRHR2** | **rs1029489** | **IL4R** | **rs12074883** | **PTGFR** |
| **rs1417938** | **CRP** | **rs1805015** | **IL4R** | **rs12725125** | **PTGFR** |
| **rs1800947** | **CRP** | **rs1805016** | **IL4R** | **rs1322931** | **PTGFR** |
| **rs3093066** | **CRP** | **rs2057768** | **IL4R** | **rs1322934** | **PTGFR** |
| **rs11571317** | **CTLA4** | **rs2234898** | **IL4R** | **rs1555541** | **PTGFR** |
| **rs16840252** | **CTLA4** | **rs2239347** | **IL4R** | **rs3766333** | **PTGFR** |
| **rs231726** | **CTLA4** | **rs3024530** | **IL4R** | **rs3766345** | **PTGFR** |
| **rs231727** | **CTLA4** | **rs3024537** | **IL4R** | **rs3766354** | **PTGFR** |
| **rs231770** | **CTLA4** | **rs3024547** | **IL4R** | **rs622346** | **PTGFR** |
| **rs231777** | **CTLA4** | **rs3024548** | **IL4R** | **rs668005** | **PTGFR** |
| **rs231779** | **CTLA4** | **rs3024560** | **IL4R** | **rs6701594** | **PTGFR** |
| **rs3087243** | **CTLA4** | **rs3024623** | **IL4R** | **rs10306135** | **PTGS1** |
| **rs5742909** | **CTLA4** | **rs3024648** | **IL4R** | **rs10306150** | **PTGS1** |
| **rs1004982** | **CYP19A1** | **rs3024658** | **IL4R** | **rs10306153** | **PTGS1** |
| **rs10163138** | **CYP19A1** | **rs3024668** | **IL4R** | **rs10306188** | **PTGS1** |
| **rs10459592** | **CYP19A1** | **rs3024675** | **IL4R** | **rs10306202** | **PTGS1** |
| **rs10519295** | **CYP19A1** | **rs3024676** | **IL4R** | **rs1213266** | **PTGS1** |
| **rs1054984** | **CYP19A1** | **rs4787423** | **IL4R** | **rs12238505** | **PTGS1** |
| **rs11856927** | **CYP19A1** | **rs4787948** | **IL4R** | **rs3842788** | **PTGS1** |
| **rs12050767** | **CYP19A1** | **rs4787956** | **IL4R** | **rs3842798** | **PTGS1** |
| **rs12591359** | **CYP19A1** | **rs6498012** | **IL4R** | **rs3842801** | **PTGS1** |
| **rs12911554** | **CYP19A1** | **rs8832** | **IL4R** | **rs4240474** | **PTGS1** |
| **rs16953058** | **CYP19A1** | **rs739718** | **IL5** | **rs4273915** | **PTGS1** |
| **rs16964201** | **CYP19A1** | **rs739719** | **IL5** | **rs7866582** | **PTGS1** |
| **rs16964258** | **CYP19A1** | **rs743562** | **IL5** | **rs9299282** | **PTGS1** |
| **rs17523880** | **CYP19A1** | **rs11766273** | **IL6** | **rs10911905** | **PTGS2** |
| **rs17523922** | **CYP19A1** | **rs12700386** | **IL6** | **rs12042763** | **PTGS2** |
| **rs17647719** | **CYP19A1** | **rs1554606** | **IL6** | **rs2066826** | **PTGS2** |
| **rs17703883** | **CYP19A1** | **rs1800795** | **IL6** | **rs2179555** | **PTGS2** |
| **rs17703982** | **CYP19A1** | **rs1800797** | **IL6** | **rs2745557** | **PTGS2** |
| **rs1902584** | **CYP19A1** | **rs1880243** | **IL6** | **rs689462** | **PTGS2** |
| **rs1902585** | **CYP19A1** | **rs2069840** | **IL6** | **rs689466** | **PTGS2** |
| **rs1902586** | **CYP19A1** | **rs10752641** | **IL6R** | **rs2476601** | **PTPN22** |
| **rs1961177** | **CYP19A1** | **rs11265610** | **IL6R** | **rs10897270** | **SCGB1A1** |
| **rs2305707** | **CYP19A1** | **rs11265618** | **IL6R** | **rs17145874** | **SCGB1A1** |
| **rs2470150** | **CYP19A1** | **rs1386821** | **IL6R** | **rs3741240** | **SCGB1A1** |
| **rs2470152** | **CYP19A1** | **rs2229238** | **IL6R** | **rs1050813** | **SERPINE1** |
| **rs2470176** | **CYP19A1** | **rs4072391** | **IL6R** | **rs11560324** | **SERPINE1** |
| **rs2899470** | **CYP19A1** | **rs4075015** | **IL6R** | **rs2070682** | **SERPINE1** |
| **rs2899472** | **CYP19A1** | **rs4329505** | **IL6R** | **rs2227631** | **SERPINE1** |
| **rs2899473** | **CYP19A1** | **rs4537545** | **IL6R** | **rs2227667** | **SERPINE1** |
| **rs3751591** | **CYP19A1** | **rs4553185** | **IL6R** | **rs2227672** | **SERPINE1** |
| **rs3751592** | **CYP19A1** | **rs4845374** | **IL6R** | **rs6950982** | **SERPINE1** |
| **rs3764221** | **CYP19A1** | **rs4845618** | **IL6R** | **rs646474** | **SERPINH1** |
| **rs3784308** | **CYP19A1** | **rs4845622** | **IL6R** | **rs649257** | **SERPINH1** |
| **rs4275794** | **CYP19A1** | **rs4845623** | **IL6R** | rs1979277 | SHMT1 |
| **rs4545755** | **CYP19A1** | **rs4845625** | **IL6R** | rs2273026 | SHMT1 |
| **rs4614671** | **CYP19A1** | **rs6687726** | **IL6R** | rs2273027 | SHMT1 |
| **rs4775932** | **CYP19A1** | **rs7526293** | **IL6R** | **rs11950646** | **SLC23A1** |
| **rs4775934** | **CYP19A1** | **rs7549338** | **IL6R** | **rs6596471** | **SLC23A1** |
| **rs4775935** | **CYP19A1** | **rs952146** | **IL6R** | **rs6596473** | **SLC23A1** |
| **rs700518** | **CYP19A1** | **rs4694178** | **IL8** | **rs1042173** | **SLC6A4** |
| **rs7172156** | **CYP19A1** | **rs4694637** | **IL8** | **rs12150214** | **SLC6A4** |
| **rs7174997** | **CYP19A1** | **rs1008562** | **IL8RA** | **rs140700** | **SLC6A4** |
| **rs726547** | **CYP19A1** | **rs2854386** | **IL8RA** | **rs140701** | **SLC6A4** |
| **rs727479** | **CYP19A1** | **rs1207362** | **KL** | **rs16965628** | **SLC6A4** |
| **rs749292** | **CYP19A1** | **rs1334928** | **KL** | **rs2020933** | **SLC6A4** |
| **rs767199** | **CYP19A1** | **rs1888057** | **KL** | **rs2020936** | **SLC6A4** |
| **rs8025191** | **CYP19A1** | **rs2149860** | **KL** | **rs2020942** | **SLC6A4** |
| **rs936306** | **CYP19A1** | **rs398655** | **KL** | **rs3794808** | **SLC6A4** |
| **rs936307** | **CYP19A1** | **rs495392** | **KL** | **rs4251417** | **SLC6A4** |
| **rs2470893** | **CYP1A1** | **rs522796** | **KL** | **rs6354** | **SLC6A4** |
| **rs5758589** | **CYP2D6** | **rs526906** | **KL** | **rs7224199** | **SLC6A4** |
| rs17466573 | DEFA3 | **rs577912** | **KL** | **rs9903602** | **SLC6A4** |
| rs7825750 | DEFA3 | **rs582524** | **KL** | **rs2267163** | **TCN2** |
| **rs10072026** | **DHFR** | **rs643780** | **KL** | rs10179730 | TFPI |
| **rs12517451** | **DHFR** | **rs648202** | **KL** | rs10187622 | TFPI |
| **rs1650697** | **DHFR** | **rs657049** | **KL** | rs12613071 | TFPI |
| **rs1650723** | **DHFR** | **rs659117** | **KL** | rs12693471 | TFPI |
| **rs1677693** | **DHFR** | **rs685417** | **KL** | rs16829086 | TFPI |
| **rs380691** | **DHFR** | **rs9526983** | **KL** | rs2041778 | TFPI |
| **rs1077218** | **EDN2** | **rs9527025** | **KL** | rs2192824 | TFPI |
| **rs11572340** | **EDN2** | **rs9536239** | **KL** | rs3213739 | TFPI |
| **rs12069358** | **EDN2** | **rs10082466** | **MBL2** | rs3755248 | TFPI |
| **rs12718439** | **EDN2** | **rs10824793** | **MBL2** | rs5940 | TFPI |
| **rs1407550** | **EDN2** | **rs10824796** | **MBL2** | rs6434222 | TFPI |
| **rs3754287** | **EDN2** | **rs11003123** | **MBL2** | rs7573488 | TFPI |
| **rs4660541** | **EDN2** | **rs11003129** | **MBL2** | rs7586970 | TFPI |
| **rs6690839** | **EDN2** | **rs12771266** | **MBL2** | rs7594359 | TFPI |
| **rs883304** | **EDN2** | **rs16933062** | **MBL2** | rs8176508 | TFPI |
| **rs1051741** | **EPHX1** | **rs1838065** | **MBL2** | rs8176541 | TFPI |
| **rs1877724** | **EPHX1** | **rs1838066** | **MBL2** | rs8176605 | TFPI |
| **rs2260863** | **EPHX1** | **rs2506** | **MBL2** | **rs10417924** | **TGFB1** |
| **rs2671272** | **EPHX1** | **rs930507** | **MBL2** | **rs1982072** | **TGFB1** |
| **rs2740168** | **EPHX1** | **rs1155764** | **MMP1** | **rs11704261** | **TIMP3** |
| **rs2740170** | **EPHX1** | **rs17293823** | **MMP1** | **rs130274** | **TIMP3** |
| **rs2854450** | **EPHX1** | **rs1939008** | **MMP1** | **rs130287** | **TIMP3** |
| **rs360063** | **EPHX1** | **rs2071230** | **MMP1** | **rs130290** | **TIMP3** |
| **rs3753658** | **EPHX1** | **rs470358** | **MMP1** | **rs130293** | **TIMP3** |
| **rs3753663** | **EPHX1** | **rs470747** | **MMP1** | **rs130300** | **TIMP3** |
| **rs1042064** | **EPHX2** | **rs484915** | **MMP1** | **rs135029** | **TIMP3** |
| **rs10503812** | **EPHX2** | **rs5031036** | **MMP1** | **rs137485** | **TIMP3** |
| **rs13269963** | **EPHX2** | **rs7125062** | **MMP1** | **rs137487** | **TIMP3** |
| **rs17057288** | **EPHX2** | **rs7945189** | **MMP1** | **rs137489** | **TIMP3** |
| **rs17057312** | **EPHX2** | **rs996999** | **MMP1** | **rs1427376** | **TIMP3** |
| **rs2640726** | **EPHX2** | **rs1053605** | **MMP2** | **rs1427378** | **TIMP3** |
| **rs2741334** | **EPHX2** | **rs11541998** | **MMP2** | **rs2040435** | **TIMP3** |
| **rs4149239** | **EPHX2** | **rs11639960** | **MMP2** | **rs2267183** | **TIMP3** |
| **rs4149252** | **EPHX2** | **rs11646643** | **MMP2** | **rs242072** | **TIMP3** |
| **rs4149253** | **EPHX2** | **rs1477017** | **MMP2** | **rs242076** | **TIMP3** |
| **rs4149259** | **EPHX2** | **rs183112** | **MMP2** | **rs242078** | **TIMP3** |
| **rs721619** | **EPHX2** | **rs1992116** | **MMP2** | **rs242082** | **TIMP3** |
| **rs7341557** | **EPHX2** | **rs2192853** | **MMP2** | **rs242089** | **TIMP3** |
| **rs7816586** | **EPHX2** | **rs2241145** | **MMP2** | **rs4504** | **TIMP3** |
| **rs891401** | **EPHX2** | **rs2241148** | **MMP2** | **rs5749524** | **TIMP3** |
| rs2026160 | F10 | **rs243831** | **MMP2** | **rs5749527** | **TIMP3** |
| rs3093261 | F10 | **rs243832** | **MMP2** | **rs5749529** | **TIMP3** |
| rs3211744 | F10 | **rs243834** | **MMP2** | **rs5754289** | **TIMP3** |
| rs3211764 | F10 | **rs243836** | **MMP2** | **rs5754312** | **TIMP3** |
| rs3211770 | F10 | **rs243842** | **MMP2** | **rs738992** | **TIMP3** |
| rs3212998 | F10 | **rs243845** | **MMP2** | **rs80272** | **TIMP3** |
| rs3213004 | F10 | **rs243866** | **MMP2** | **rs9606994** | **TIMP3** |
| rs474810 | F10 | **rs8054459** | **MMP2** | **rs9619311** | **TIMP3** |
| rs547138 | F10 | **rs865094** | **MMP2** | **rs9862** | **TIMP3** |
| rs559054 | F10 | **rs866770** | **MMP2** | **rs3755724** | **TIMP4** |
| rs5960 | F10 | **rs9922534** | **MMP2** | **rs3773364** | **TIMP4** |
| rs6046 | F10 | **rs520540** | **MMP3** | **rs4684841** | **TIMP4** |
| rs9549675 | F10 | **rs522616** | **MMP3** | **rs99365** | **TIMP4** |
| **rs2070852** | **F2** | **rs569444** | **MMP3** | **rs1337** | **TLR2** |
| **rs3136485** | **F2** | **rs645419** | **MMP3** | **rs1898830** | **TLR2** |
| rs11954573 | F2R | **rs650108** | **MMP3** | **rs4696483** | **TLR2** |
| rs153311 | F2R | **rs10895354** | **MMP8** | **rs7656411** | **TLR2** |
| rs2227744 | F2R | **rs11225394** | **MMP8** | **rs11721827** | **TLR3** |
| rs2227827 | F2R | **rs1276284** | **MMP8** | **rs3775291** | **TLR3** |
| rs250731 | F2R | **rs17099443** | **MMP8** | **rs3775292** | **TLR3** |
| rs250738 | F2R | **rs1939020** | **MMP8** | **rs4862632** | **TLR3** |
| rs27593 | F2R | **rs1940475** | **MMP8** | **rs4862633** | **TLR3** |
| rs37249 | F2R | **rs2508383** | **MMP8** | **rs5743303** | **TLR3** |
| rs2242991 | F2RL1 | **rs6590985** | **MMP8** | **rs5743305** | **TLR3** |
| rs2243004 | F2RL1 | **rs4810482** | **MMP9** | **rs5743312** | **TLR3** |
| rs2243010 | F2RL1 | **rs6104420** | **MMP9** | **rs7668666** | **TLR3** |
| rs2243066 | F2RL1 | **rs8113877** | **MMP9** | **rs10759930** | **TLR4** |
| rs2243083 | F2RL1 | **rs11849530** | **MTHFD1** | **rs10759932** | **TLR4** |
| rs34308580 | F2RL1 | **rs1256146** | **MTHFD1** | **rs11536889** | **TLR4** |
| rs631465 | F2RL1 | **rs17751556** | **MTHFD1** | **rs11536898** | **TLR4** |
| rs639342 | F2RL1 | **rs17824591** | **MTHFD1** | **rs1554973** | **TLR4** |
| rs6453251 | F2RL1 | **rs1885031** | **MTHFD1** | **rs16906053** | **TLR4** |
| rs6453253 | F2RL1 | **rs1950902** | **MTHFD1** | **rs1927906** | **TLR4** |
| rs1054533 | F2RL3 | **rs1956545** | **MTHFD1** | **rs1927911** | **TLR4** |
| rs2227356 | F2RL3 | **rs2236225** | **MTHFD1** | **rs2149356** | **TLR4** |
| rs2608732 | F2RL3 | **rs2295640** | **MTHFD1** | **rs2770150** | **TLR4** |
| rs773901 | F2RL3 | **rs3783731** | **MTHFD1** | **rs7856729** | **TLR4** |
| rs28672143 | F3 | **rs3818239** | **MTHFD1** | **rs7869402** | **TLR4** |
| rs696619 | F3 | **rs745686** | **MTHFD1** | **rs1731478** | **TLR7** |
| rs762484 | F3 | **rs8016556** | **MTHFD1** | **rs179006** | **TLR7** |
| rs762485 | F3 | **rs11121832** | **MTHFR** | **rs179007** | **TLR7** |
| **rs10489185** | **F5** | **rs12121543** | **MTHFR** | **rs179008** | **TLR7** |
| **rs12120605** | **F5** | **rs1476413** | **MTHFR** | **rs179009** | **TLR7** |
| **rs12131397** | **F5** | **rs17367504** | **MTHFR** | **rs179011** | **TLR7** |
| **rs12755775** | **F5** | **rs17421462** | **MTHFR** | **rs179012** | **TLR7** |
| **rs1557572** | **F5** | **rs17421511** | **MTHFR** | **rs179016** | **TLR7** |
| **rs1894697** | **F5** | **rs1801131** | **MTHFR** | **rs179021** | **TLR7** |
| **rs2187952** | **F5** | **rs1801133** | **MTHFR** | **rs5741880** | **TLR7** |
| **rs2213865** | **F5** | **rs1994798** | **MTHFR** | **rs5743740** | **TLR7** |
| **rs2298908** | **F5** | **rs3737964** | **MTHFR** | **rs5935438** | **TLR7** |
| **rs2420369** | **F5** | **rs3753582** | **MTHFR** | **rs864058** | **TLR7** |
| **rs3766103** | **F5** | **rs4846048** | **MTHFR** | **rs16987224** | **TLR8** |
| **rs3917854** | **F5** | **rs4846049** | **MTHFR** | **rs17256081** | **TLR8** |
| **rs4656687** | **F5** | **rs4846052** | **MTHFR** | **rs2159377** | **TLR8** |
| **rs6019** | **F5** | **rs9651118** | **MTHFR** | **rs2407992** | **TLR8** |
| **rs6020** | **F5** | rs12759827 | MTR | **rs3747414** | **TLR8** |
| **rs6022** | **F5** | rs1805087 | MTR | **rs3761621** | **TLR8** |
| **rs6035** | **F5** | rs4077829 | MTR | **rs3761623** | **TLR8** |
| **rs6427197** | **F5** | rs4659723 | MTR | **rs3764880** | **TLR8** |
| **rs6427198** | **F5** | rs4659743 | MTR | **rs5741883** | **TLR8** |
| **rs9287095** | **F5** | rs10380 | MTRR | **rs5741886** | **TLR8** |
| **rs9332575** | **F5** | rs1532268 | MTRR | **rs5741890** | **TLR8** |
| **rs9332618** | **F5** | rs162031 | MTRR | **rs5744068** | **TLR8** |
| **rs9332624** | **F5** | rs162033 | MTRR | **rs352143** | **TLR9** |
| **rs1475931** | **F7** | rs162036 | MTRR | **rs1799964** | **TNF** |
| **rs3211719** | **F7** | rs16879258 | MTRR | **rs2229094** | **TNF** |
| **rs488703** | **F7** | rs17184211 | MTRR | **rs2844482** | **TNF** |
| **rs555212** | **F7** | rs1801394 | MTRR | **rs1800693** | **TNFRSF1A** |
| **rs1051070** | **FAS** | rs326121 | MTRR | **rs1860545** | **TNFRSF1A** |
| **rs1571011** | **FAS** | rs326124 | MTRR | **rs2302350** | **TNFRSF1A** |
| **rs2031611** | **FAS** | rs3815743 | MTRR | **rs3764874** | **TNFRSF1A** |
| **rs2234978** | **FAS** | rs7703033 | MTRR | **rs4149570** | **TNFRSF1A** |
| **rs3758483** | **FAS** | rs7730643 | MTRR | **rs4149577** | **TNFRSF1A** |
| **rs4934434** | **FAS** | **rs10888150** | **NAT1** | **rs4149578** | **TNFRSF1A** |
| **rs6586165** | **FAS** | **rs17126350** | **NAT1** | **rs4149622** | **TNFRSF1A** |
| **rs7901656** | **FAS** | **rs4298522** | **NAT1** | **rs740841** | **TNFRSF1A** |
| **rs7915235** | **FAS** | **rs4921880** | **NAT1** | **rs1061622** | **TNFRSF1B** |
| **rs9658727** | **FAS** | **rs7003890** | **NAT1** | **rs1061624** | **TNFRSF1B** |
| **rs9658742** | **FAS** | **rs7017402** | **NAT1** | **rs1061628** | **TNFRSF1B** |
| **rs9658761** | **FAS** | **rs8190837** | **NAT1** | **rs1061631** | **TNFRSF1B** |
| **rs982764** | **FAS** | **rs8190870** | **NAT1** | **rs1201157** | **TNFRSF1B** |
| **rs983751** | **FAS** | **rs9325827** | **NAT1** | **rs235214** | **TNFRSF1B** |
| **rs12041613** | **FASLG** | **rs1208** | **NAT2** | **rs235219** | **TNFRSF1B** |
| **rs17370527** | **FASLG** | **rs1799929** | **NAT2** | **rs3766730** | **TNFRSF1B** |
| **rs2639614** | **FASLG** | **rs1801280** | **NAT2** | **rs474247** | **TNFRSF1B** |
| **rs2859242** | **FASLG** | **rs4646246** | **NAT2** | **rs522807** | **TNFRSF1B** |
| **rs5030772** | **FASLG** | **rs721398** | **NAT2** | **rs5746051** | **TNFRSF1B** |
| **rs6700734** | **FASLG** | **rs721399** | **NAT2** | **rs5746053** | **TNFRSF1B** |
| **rs947895** | **GSTP1** | **rs7832071** | **NAT2** | **rs590368** | **TNFRSF1B** |
| **rs11799643** | **HSD11B1** | **rs10489113** | **NFKB1** | **rs616645** | **TNFRSF1B** |
| **rs12040780** | **HSD11B1** | **rs13117745** | **NFKB1** | **rs652625** | **TNFRSF1B** |
| **rs17389016** | **HSD11B1** | **rs1585213** | **NFKB1** | **rs816050** | **TNFRSF1B** |
| **rs2235543** | **HSD11B1** | **rs1599961** | **NFKB1** | **rs976881** | **TNFRSF1B** |
| **rs3753519** | **HSD11B1** | **rs1609798** | **NFKB1** | **rs2784075** | **TRAF2** |
| **rs4844880** | **HSD11B1** | **rs1801** | **NFKB1** | **rs908831** | **TRAF2** |
| **rs6672256** | **HSD11B1** | **rs230528** | **NFKB1** | **rs1385105** | **TREM1** |
| **rs846910** | **HSD11B1** | **rs3755867** | **NFKB1** | **rs16894387** | **TREM1** |
| **rs846911** | **HSD11B1** | **rs3774933** | **NFKB1** | **rs1817537** | **TREM1** |
| **rs932335** | **HSD11B1** | **rs3817685** | **NFKB1** | **rs2234243** | **TREM1** |
| **rs9430012** | **HSD11B1** | **rs4648058** | **NFKB1** | **rs3804277** | **TREM1** |
| **rs1039874** | **HSD17B7** | **rs4648090** | **NFKB1** | **rs3827632** | **TREM1** |
| **rs11589262** | **HSD17B7** | **rs4648135** | **NFKB1** | **rs4711668** | **TREM1** |
| **rs1780019** | **HSD17B7** | **rs4648141** | **NFKB1** | **rs6910730** | **TREM1** |
| **rs2803865** | **HSD17B7** | **rs7674640** | **NFKB1** | **rs6939973** | **TREM1** |
| **rs2805053** | **HSD17B7** | **rs980455** | **NFKB1** | **rs6940092** | **TREM1** |
| **rs4656381** | **HSD17B7** | **rs997476** | **NFKB1** | **rs1005292** | **TSHR** |
| **rs10906772** | **HSPA14** | **rs1056890** | **NFKB2** | **rs10129380** | **TSHR** |
| **rs10906774** | **HSPA14** | **rs11574845** | **NFKB2** | **rs1035145** | **TSHR** |
| **rs11593057** | **HSPA14** | **rs7897947** | **NFKB2** | **rs10483973** | **TSHR** |
| **rs17155992** | **HSPA14** | **rs3138045** | **NFKBIA** | **rs11159491** | **TSHR** |
| **rs7894284** | **HSPA14** | **rs696** | **NFKBIA** | **rs11845715** | **TSHR** |
| **rs7905174** | **HSPA14** | **rs11083487** | **NFKBIB** | **rs12881268** | **TSHR** |
| **rs9787671** | **HSPA14** | **rs11575002** | **NFKBIB** | **rs12883801** | **TSHR** |
| **rs1043618** | **HSPA1A** | **rs2053071** | **NFKBIB** | **rs12885526** | **TSHR** |
| **rs2471980** | **HSPA1B** | **rs2241704** | **NFKBIB** | **rs12892567** | **TSHR** |
| **rs2763979** | **HSPA1B** | **rs2241705** | **NFKBIB** | **rs17111361** | **TSHR** |
| **rs2075800** | **HSPA1L** | **rs3136641** | **NFKBIB** | **rs17111394** | **TSHR** |
| **rs2227956** | **HSPA1L** | **rs3136646** | **NFKBIB** | **rs17111431** | **TSHR** |
| **rs10075878** | **HSPA4** | **rs1875324** | **NFKBIE** | **rs17111481** | **TSHR** |
| **rs4574536** | **HSPA4** | **rs2282151** | **NFKBIE** | **rs17111530** | **TSHR** |
| **rs4616886** | **HSPA4** | **rs483536** | **NFKBIE** | **rs17545310** | **TSHR** |
| **rs4705990** | **HSPA4** | **rs520639** | **NFKBIE** | **rs17630128** | **TSHR** |
| **rs7730747** | **HSPA4** | **rs730775** | **NFKBIE** | **rs179247** | **TSHR** |
| **rs12129787** | **HSPA6** | **rs10277237** | **NOS3** | **rs179259** | **TSHR** |
| **rs2099684** | **HSPA6** | **rs12703107** | **NOS3** | **rs179260** | **TSHR** |
| **rs404508** | **HSPA6** | **rs1799983** | **NOS3** | **rs179261** | **TSHR** |
| **rs4657054** | **HSPA6** | **rs1800783** | **NOS3** | **rs1957547** | **TSHR** |
| **rs9427401** | **HSPA6** | **rs2373929** | **NOS3** | **rs1991517** | **TSHR** |
| **rs10878763** | **IFNG** | **rs3918227** | **NOS3** | **rs2024426** | **TSHR** |
| **rs2069705** | **IFNG** | **rs743507** | **NOS3** | **rs2075173** | **TSHR** |
| **rs2069716** | **IFNG** | **rs10482682** | **NR3C1** | **rs2110696** | **TSHR** |
| **rs2069718** | **IFNG** | **rs12655166** | **NR3C1** | **rs2110697** | **TSHR** |
| **rs2069727** | **IFNG** | **rs12656106** | **NR3C1** | **rs2268451** | **TSHR** |
| **rs1520220** | **IGF1** | **rs13182800** | **NR3C1** | **rs2268466** | **TSHR** |
| **rs2471551** | **IGFBP3** | **rs17100236** | **NR3C1** | **rs2268474** | **TSHR** |
| **rs1800872** | **IL10** | **rs17287758** | **NR3C1** | **rs2268475** | **TSHR** |
| **rs1800890** | **IL10** | **rs2918417** | **NR3C1** | **rs2268476** | **TSHR** |
| **rs1800896** | **IL10** | **rs2963155** | **NR3C1** | **rs2268477** | **TSHR** |
| **rs3024496** | **IL10** | **rs33388** | **NR3C1** | **rs2284735** | **TSHR** |
| **rs3024498** | **IL10** | **rs4244032** | **NR3C1** | **rs2288493** | **TSHR** |
| **rs11216666** | **IL10RA** | **rs4607376** | **NR3C1** | **rs2300520** | **TSHR** |
| **rs17121510** | **IL10RA** | **rs4634384** | **NR3C1** | **rs2300521** | **TSHR** |
| **rs2229113** | **IL10RA** | **rs4912911** | **NR3C1** | **rs2300525** | **TSHR** |
| **rs2508445** | **IL10RA** | **rs4986593** | **NR3C1** | **rs2300528** | **TSHR** |
| **rs2512143** | **IL10RA** | **rs7701443** | **NR3C1** | **rs2300540** | **TSHR** |
| **rs4252254** | **IL10RA** | **rs9324918** | **NR3C1** | **rs3783938** | **TSHR** |
| **rs4252270** | **IL10RA** | **rs9324921** | **NR3C1** | **rs3783943** | **TSHR** |
| **rs4936414** | **IL10RA** | **rs9324924** | **NR3C1** | **rs3783948** | **TSHR** |
| **rs4938467** | **IL10RA** | **rs1029744** | **PAFAH1B1** | **rs3783950** | **TSHR** |
| **rs947889** | **IL10RA** | **rs11078302** | **PAFAH1B1** | **rs4903964** | **TSHR** |
| **rs9610** | **IL10RA** | **rs12938775** | **PAFAH1B1** | **rs4903967** | **TSHR** |
| **rs1058867** | **IL10RB** | **rs2317297** | **PAFAH1B1** | **rs6574616** | **TSHR** |
| **rs2243498** | **IL10RB** | **rs3785958** | **PAFAH1B1** | **rs6574629** | **TSHR** |
| **rs2284552** | **IL10RB** | **rs4790353** | **PAFAH1B1** | **rs7143071** | **TSHR** |
| **rs2834168** | **IL10RB** | **rs4790355** | **PAFAH1B1** | **rs7144481** | **TSHR** |
| **rs2834170** | **IL10RB** | **rs4790356** | **PAFAH1B1** | **rs7157900** | **TSHR** |
| **rs2834172** | **IL10RB** | **rs6502385** | **PAFAH1B1** | **rs7158881** | **TSHR** |
| **rs2834175** | **IL10RB** | **rs7209407** | **PAFAH1B1** | **rs7161100** | **TSHR** |
| **rs6517158** | **IL10RB** | **rs7213463** | **PAFAH1B1** | **rs722540** | **TSHR** |
| **rs765429** | **IL10RB** | **rs7223411** | **PAFAH1B1** | **rs724169** | **TSHR** |
| **rs999259** | **IL10RB** | **rs3736120** | **PAFAH1B2** | **rs8009058** | **TSHR** |
| **rs999261** | **IL10RB** | **rs4938347** | **PAFAH1B2** | **rs8012937** | **TSHR** |
| **rs1295683** | **IL13** | **rs5757231** | **PGEA1** | **rs8017455** | **TSHR** |
| **rs1295686** | **IL13** | **rs6001188** | **PGEA1** | **rs917984** | **TSHR** |
| **rs2243204** | **IL13** | **rs6001193** | **PGEA1** | **rs917986** | **TSHR** |
| **rs3091307** | **IL13** | **rs6519132** | **PGEA1** | **rs930099** | **TSHR** |
| **rs848** | **IL13** | **rs11224561** | **PGR** | **rs10929303** | **UGT1A1** |
| **rs12498901** | **IL15** | **rs11224575** | **PGR** | **rs11888492** | **UGT1A1** |
| **rs12508866** | **IL15** | **rs11224589** | **PGR** | **rs1500477** | **UGT1A1** |
| **rs13117878** | **IL15** | **rs471767** | **PGR** | **rs1500482** | **UGT1A1** |
| **rs1519551** | **IL15** | **rs492457** | **PGR** | **rs2302538** | **UGT1A1** |
| **rs1519552** | **IL15** | **rs503362** | **PGR** | **rs3755319** | **UGT1A1** |
| **rs17007610** | **IL15** | **rs504372** | **PGR** | **rs4148324** | **UGT1A1** |
| **rs17461269** | **IL15** | **rs507141** | **PGR** | **rs4148328** | **UGT1A1** |
| **rs1907949** | **IL15** | **rs518162** | **PGR** | **rs4148329** | **UGT1A1** |
| **rs6537061** | **IL15** | **rs518382** | **PGR** | **rs4663972** | **UGT1A1** |
| **rs6850492** | **IL15** | **rs537681** | **PGR** | **rs6717546** | **UGT1A1** |
| **rs7698675** | **IL15** | **rs538915** | **PGR** | **rs6719561** | **UGT1A1** |
| **rs1293344** | **IL18** | **rs542384** | **PGR** | **rs6742078** | **UGT1A1** |
| **rs1946519** | **IL18** | **rs553272** | **PGR** | **rs7586006** | **UGT1A1** |
| **rs2043055** | **IL18** | **rs555572** | **PGR** | **rs8330** | **UGT1A1** |
| **rs360722** | **IL18** | **rs563656** | **PGR** | **rs929596** | **UGT1A1** |
| **rs4937113** | **IL18** | **rs578029** | **PGR** | **rs3025010** | **VEGF** |
| **rs543810** | **IL18** | **rs619487** | **PGR** | **rs3025033** | **VEGF** |
| **rs5744222** | **IL18** | **rs635984** | **PGR** | **rs3025035** | **VEGF** |
| **rs5744280** | **IL18** | **rs653752** | **PGR** | **rs6900017** | **VEGF** |
| **rs17561** | **IL1A** | **rs660149** | **PGR** | **rs699947** | **VEGF** |
| **rs1878321** | **IL1A** | **rs2428757** | **PGRMC1** | **rs833068** | **VEGF** |
| **rs2856838** | **IL1A** | **rs2499043** | **PGRMC1** | **rs833069** | **VEGF** |
| **rs1143623** | **IL1B** | **rs11726595** | **PGRMC2** | **rs998584** | **VEGF** |
| **rs1143627** | **IL1B** | **rs2036687** | **PGRMC2** |  |  |

Bolded SNPs are those that overlap with Cenn study.
