# Supplementary material for: Maternal and Fetal Genetic Associations of PTGER3 and PON1 with Preterm Birth
Source: PLoS One. 2010 Feb 3;5(2):e9040. doi: 10.1371/journal.pone.0009040 (PMC2815792; doi:10.1371/journal.pone.0009040)
Supplement: Table S2 — (0.13 MB DOC) [file pone.0009040.s004.doc]

Supplemental Table 2: Additional single locus results in Cenn study.

| Chr | Gene | SNP (RS#) | Minor  Allele | Maternal  Case MAF | Maternal Control MAF | Maternal Allele p | Maternal Genotype p | Fetal Case MAF | Fetal Control MAF | Fetal Allele p | Fetal Genotype p |
| --- | --- | --- | --- | --- | --- | --- | --- | --- | --- | --- | --- |
| 2 | TFPI | rs10179730 | G | 0.14 | 0.11 | 0.29 | 0.44 | 0.13 | 0.13 | 0.99 | 0.29 |
| 2 | TFPI | rs10187622 | T | 0.18 | 0.16 | 0.54 | 0.74 | 0.16 | 0.16 | 0.93 | 0.78 |
| 2 | TFPI | rs12613071 | C | 0.24 | 0.21 | 0.38 | 0.13 | 0.22 | 0.22 | 0.98 | 0.15 |
| 2 | TFPI | rs12693471 | G | 0.31 | 0.29 | 0.65 | 0.56 | 0.31 | 0.29 | 0.66 | 0.91 |
| 2 | TFPI | rs16829086 | T | 0.25 | 0.23 | 0.53 | 0.23 | 0.24 | 0.25 | 0.89 | 0.87 |
| 2 | TFPI | rs2041778 | C | 0.42 | 0.37 | 0.23 | 0.29 | 0.39 | 0.37 | 0.64 | 0.62 |
| 2 | TFPI | rs2192824 | T | 0.40 | 0.42 | 0.68 | 0.46 | 0.42 | 0.41 | 0.94 | 0.12 |
| 2 | TFPI | rs3213739 | T | 0.46 | 0.43 | 0.39 | 0.67 | 0.44 | 0.43 | 0.85 | 0.98 |
| 2 | TFPI | rs3755248 | G | 0.36 | 0.31 | 0.20 | 0.34 | 0.32 | 0.31 | 0.81 | 0.76 |
| 2 | TFPI | rs6434222 | A | 0.11 | 0.12 | 0.92 | 1.00 | 0.10 | 0.10 | 0.93 | 1.00 |
| 2 | TFPI | rs7573488 | C | 0.26 | 0.24 | 0.53 | 0.60 | 0.26 | 0.24 | 0.64 | 0.86 |
| 2 | TFPI | rs7586970 | C | 0.32 | 0.30 | 0.64 | 0.41 | 0.31 | 0.30 | 0.93 | 1.00 |
| 2 | TFPI | rs7594359 | T | 0.43 | 0.48 | 0.21 | 0.36 | 0.48 | 0.48 | 0.98 | 0.71 |
| 2 | TFPI | rs8176508 | T | 0.34 | 0.36 | 0.62 | 0.51 | 0.36 | 0.37 | 0.91 | 0.22 |
| 2 | TFPI | rs8176541 | T | 0.31 | 0.29 | 0.55 | 0.43 | 0.31 | 0.29 | 0.66 | 0.91 |
| 5 | MTRR | rs10380 | T | 0.12 | 0.11 | 0.72 | 0.88 | 0.10 | 0.11 | 0.64 | 0.93 |
| 5 | MTRR | rs1532268 | A | 0.31 | 0.35 | 0.35 | 0.62 | 0.35 | 0.38 | 0.45 | 0.47 |
| 5 | MTRR | rs162031 | T | 0.28^1^ | 0.23^2^ | 0.16 | 0.30 | 0.25^1^ | 0.28^2^ | 0.54 | 0.21 |
| 5 | MTRR | rs162033 | T | 0.44 | 0.45 | 0.93 | 0.17 | 0.45 | 0.39 | 0.22 | 0.22 |
| 5 | MTRR | rs162036 | G | 0.14 | 0.13 | 0.74 | 0.89 | 0.13 | 0.12 | 0.73 | 0.74 |
| 5 | MTRR | rs326124 | A | 0.17 | 0.14 | 0.19 | 0.19 | 0.16 | 0.18 | 0.57 | 0.77 |
| 5 | MTRR | rs3815743 | G | 0.12 | 0.16 | 0.23 | 0.53 | 0.15 | 0.17 | 0.51 | 0.71 |
| 5 | MTRR | rs7703033 | A | 0.31 | 0.34 | 0.44 | 0.57 | 0.32 | 0.29 | 0.37 | 0.46 |
| 5 | MTRR | rs7730643 | G | 0.16 | 0.16 | 0.88 | 0.67 | 0.18 | 0.16 | 0.53 | 0.65 |
| 6 | PLG | rs11060 | T | 0.44 | 0.45 | 0.87 | 0.57 | 0.47 | 0.45 | 0.58 | 0.72 |
| 6 | PLG | rs1950562 | A | 0.43 | 0.44 | 0.80 | 0.86 | 0.42 | 0.46 | 0.38 | 0.54 |
| 6 | PLG | rs4252092 | A | 0.43 | 0.41 | 0.60 | 0.85 | 0.40 | 0.44 | 0.29 | 0.46 |
| 6 | PLG | rs4252125 | A | 0.25 | 0.28 | 0.43 | 0.66 | 0.27^1^ | 0.28 | 0.86 | 0.16 |
| 6 | PLG | rs4252159 | A | 0.08 | 0.10^2^ | 0.40 | 0.46 | 0.07 | 0.06 | 0.39 | 0.48 |
| 6 | PLG | rs4252166 | C | 0.14 | 0.18 | 0.20 | 0.49 | 0.17 | 0.18 | 0.57 | 0.82 |
| 6 | PLG | rs783144 | A | 0.29 | 0.26 | 0.40 | 0.28 | 0.29 | 0.29 | 1.00 | 0.22 |
| 6 | PLG | rs783147 | T | 0.40 | 0.46 | 0.14 | 0.12 | 0.42^1^ | 0.41 | 0.85 | 0.17 |
| 6 | PLG | rs783166 | T | 0.15 | 0.09 | 0.02 | 0.02 | 0.13 | 0.09^2^ | 0.10 | 3.5E-03 |
| 6 | PLG | rs783176 | C | 0.17 | 0.18 | 0.64 | 0.85 | 0.17 | 0.15 | 0.52 | 0.68 |
| 6 | PLG | rs813641 | T | 0.16 | 0.19 | 0.29^2^ | 0.52 | 0.15 | 0.14 | 0.74 | 0.63 |
| 6 | PLG | rs9295131 | G | 0.32 | 0.31 | 0.80^2^ | 0.68 | 0.33 | 0.27 | 0.16 | 0.40 |
| 6 | PLG | rs9458011 | T | 0.04 | 0.06 | 0.24 | 0.25 | 0.05 | 0.06 | 0.35 | 0.44 |
| 6 | PLG | rs9458023 | A | 0.38 | 0.35 | 0.48 | 0.73 | 0.35 | 0.39 | 0.33 | 0.61 |

^1^Cases deviate from HWE in maternal samples at rs162031 (p = 4.9x10^-4^) and fetal samples at rs162031 (p = 1.1x10^-5^), rs4252125 (p = 3.3x10^-3^), rs783147 (p = 0.05)

^2^Controls deviate from HWE in maternal samples at rs162031 (p = 0.01), rs4252159 (p = 3.9x10^-3^), rs813641 (p = 0.01), rs9295131 (p = 9.9x10^-3^) and fetal samples at rs162031 (p = 5.3x10^-3^), rs783166 (p = 3.4x10^-4^)
