# Supplementary material for: Maternal and Fetal Genetic Associations of PTGER3 and PON1 with Preterm Birth
Source: PLoS One. 2010 Feb 3;5(2):e9040. doi: 10.1371/journal.pone.0009040 (PMC2815792; doi:10.1371/journal.pone.0009040)
Supplement: Table S3 — (0.32 MB DOC) [file pone.0009040.s005.doc]

Supplemental Table 3: Suggestive maternal and fetal single locus results in MoBa study. Left panel is maternal associations, right panel is fetal associations.

| *Maternal Gene* | *RS #* | *Allele p* | *Genotype p* | *Fetal Gene* | *RS #* | *Allele p* | *Genotype p* |
| --- | --- | --- | --- | --- | --- | --- | --- |
| ADH1B | rs17033 | 0.09 | 0.02 | ADH1B | rs17033 | 0.04 | 0.05 |
| ADRB2 | rs1432622 | 0.05 | 0.13 | COL1A2 | rs388625 | 0.01 | 0.01 |
| ADRB2 | rs12654778 | 0.03 | 0.09 | COL1A2 | rs420257 | 1.40E-03 | 9.60E-04 |
| CARD15 | rs8056611 | 0.11 | 0.04 | COL1A2 | rs389328 | 0.01 | 0.01 |
| CBS | rs6586282 | 0.22 | 4.7E-03 | COL1A2 | rs42524 | 0.01 | 0.02 |
| CBS | rs11203172 | 0.32 | 0.05 | COL1A2 | rs2521205 | 0.04 | 0.13 |
| COL1A1 | rs1061237 | 0.33 | 0.04 | COL1A2 | rs42528 | 0.01 | 0.02 |
| COL1A2 | rs2521205 | 0.02 | 0.05 | COL1A2 | rs2472 | 0.01 | 0.01 |
| COL1A2 | rs2472 | 1.6E-03 | 1.2E-03 | COL1A2 | rs441051 | 2.80E-03 | 0.01 |
| COL1A2 | rs7804898 | 0.21 | 0.02 | COL1A2 | rs411717 | 0.01 | 0.01 |
| COL3A1 | rs2271682 | 0.04 | 0.05 | COL3A1 | rs2138533 | 0.32 | 0.01 |
| COL5A1 | rs12005720 | 0.28 | 0.04 | COL5A1 | rs3124932 | 0.03 | 0.06 |
| COL5A1 | rs4842167 | 0.01 | 0.04 | COL5A2 | rs7420331 | 0.07 | 0.01 |
| COL5A1 | rs3811161 | 0.01 | 0.04 | CRH | rs6996265 | 0.03 | 0.01 |
| COL5A1 | rs10745387 | 0.03 | 0.10 | CRH | rs3176921 | 0.03 | 0.01 |
| COL5A1 | rs13946 | 0.04 | 0.12 | CRH | rs6472257 | 0.02 | 4.20E-03 |
| CRHR2 | rs4722999 | 5.0E-03 | 0.02 | CRHBP | rs10514082 | 0.34 | 0.01 |
| CRHR2 | rs12701020 | 0.09 | 0.02 | CRHR2 | rs2190242 | 0.82 | 0.05 |
| CRHR2 | rs929377 | 0.01 | 0.03 | CYP19A1 | rs8025191 | 0.04 | 0.05 |
| CRHR2 | rs2190242 | 0.03 | 0.09 | EPHX2 | rs4149239 | 0.58 | 0.03 |
| CRHR2 | rs2284219 | 0.01 | 0.04 | EPHX2 | rs891401 | 0.57 | 0.03 |
| CYP19A1 | rs727479 | 0.69 | 0.05 | EPHX2 | rs10503812 | 0.57 | 0.03 |
| CYP19A1 | rs17647719 | 0.04 | 0.08 | EPHX2 | rs4149252 | 0.58 | 0.03 |
| CYP19A1 | rs3751592 | 0.43 | 0.03 | EPHX2 | rs4149259 | 0.42 | 0.02 |
| EDN2 | rs4660541 | 0.86 | 0.05 | F2R | rs27593 | 0.33 | 0.04 |
| EPHX1 | rs2740168 | 0.85 | 0.02 | F5 | rs1557572 | 0.54 | 0.05 |
| F10 | rs3211744 | 0.07 | 0.05 | HSD11B1 | rs17389016 | 0.01 | 0.02 |
| F3 | rs762484 | 0.01 | 0.04 | HSD11B1 | rs3753519 | 0.01 | 1.50E-03 |
| F3 | rs696619 | 0.05 | 0.09 | HSD17B7 | rs4656381 | 0.03 | 2.90E-03 |
| F7 | rs1475931 | 0.46 | 2.3E-03 | HSPA1B | rs2471980 | 0.02 | 3.80E-03 |
| FAS | rs9658742 | 0.62 | 0.03 | HSPA1B | rs2763979 | 0.03 | 0.01 |
| GSTP1 | rs947895 | 0.07 | 0.04 | HSPA6 | rs9427401 | 0.01 | 0.02 |
| IL18 | rs543810 | 0.31 | 0.04 | IL10RB | rs999261 | 0.01 | 0.03 |
| IL1B | rs1143630 | 0.03 | 0.04 | IL10RB | rs6517158 | 0.04 | 0.09 |
| IL1R1 | rs3917273 | 0.04 | 0.02 | IL13 | rs1295683 | 0.02 | 0.04 |
| IL1R1 | rs2110726 | 0.43 | 0.03 | IL15 | rs17461269 | 0.03 | 0.01 |
| IL1R2 | rs11884283 | 0.17 | 0.02 | IL15 | rs1519552 | 0.02 | 0.06 |
| IL1R2 | rs12467316 | 0.06 | 0.01 | IL15 | rs7698675 | 0.03 | 0.07 |
| IL1R2 | rs1108338 | 0.02 | 0.01 | IL15 | rs13117878 | 0.02 | 0.05 |
| IL1RAP | rs7628333 | 1.8E-03 | 4.7E-03 | IL15 | rs6850492 | 0.04 | 0.09 |
| IL1RAP | rs3821744 | 4.7E-03 | 0.01 | IL15 | rs17007610 | 0.03 | 0.07 |
| IL1RAP | rs9883249 | 0.03 | 0.07 | IL15 | rs6537061 | 0.01 | 0.03 |
| IL1RN | rs315920 | 4.9E-03 | 0.01 | IL1A | rs17561 | 0.02 | 0.06 |
| IL1RN | rs4251961 | 0.03 | 0.08 | IL1A | rs2856838 | 0.03 | 0.02 |
| IL1RN | rs315946 | 0.01 | 0.03 | IL1A | rs1878321 | 0.02 | 0.06 |
| IL2RA | rs11598648 | 0.25 | 0.02 | IL1R1 | rs3917225 | 0.03 | 0.08 |
| IL2RA | rs1107345 | 0.04 | 0.06 | IL1R1 | rs2287047 | 0.01 | 0.04 |
| IL2RA | rs11256497 | 0.03 | 0.10 | IL1R1 | rs3917273 | 0.01 | 0.03 |
| IL2RA | rs706778 | 0.05 | 0.11 | IL1R1 | rs2160227 | 0.02 | 0.08 |
| IL2RA | rs3134883 | 0.04 | 0.10 | IL1R1 | rs3917304 | 0.02 | 0.07 |
| IL2RB | rs228954 | 0.01 | 0.03 | IL1RAP | rs7628333 | 0.18 | 0.02 |
| IL2RB | rs228957 | 0.01 | 0.03 | IL1RAP | rs2193880 | 0.03 | 0.08 |
| IL2RB | rs2281094 | 0.03 | 0.08 | IL1RAP | rs9877268 | 0.24 | 0.05 |
| IL4R | rs3024548 | 0.13 | 0.04 | IL1RAP | rs759783 | 0.03 | 0.09 |
| IL4R | rs3024623 | 0.05 | 0.02 | IL1RAP | rs4140711 | 0.03 | 0.09 |
| IL6R | rs4845374 | 0.04 | 0.03 | IL1RAP | rs1015704 | 0.02 | 0.04 |
| IL6R | rs4329505 | 0.04 | 0.03 | IL1RAP | rs1015705 | 0.03 | 0.09 |
| IL8RA | rs1008562 | 0.05 | 0.04 | IL1RAP | rs4687163 | 0.03 | 0.09 |
| KL | rs495392 | 0.85 | 0.02 | IL1RAP | rs929729 | 0.04 | 0.1 |
| KL | rs522796 | 0.16 | 0.04 | IL2RA | rs12722596 | 0.03 | 0.08 |
| MMP2 | rs1053605 | 0.04 | 0.07 | IL2RA | rs11256497 | 0.01 | 0.03 |
| MTHFD1 | rs17824591 | 0.03 | 0.11 | IL2RA | rs2476491 | 0.03 | 0.02 |
| MTHFD1 | rs2236225 | 0.97 | 0.04 | IL2RA | rs706778 | 0.03 | 0.09 |
| MTHFR | rs11121832 | 0.03 | 0.05 | IL2RA | rs3134883 | 0.04 | 0.09 |
| MTHFR | rs3737964 | 0.02 | 0.03 | IL4 | rs2070874 | 0.02 | 0.03 |
| MTRR | rs162031 | 0.02 | 0.05 | IL4 | rs2227284 | 0.01 | 0.03 |
| MTRR | rs3815743 | 0.03 | 0.01 | IL4 | rs2243268 | 0.02 | 0.04 |
| NAT1 | rs7017402 | 0.04 | 0.05 | IL4 | rs2243274 | 0.02 | 0.03 |
| NAT1 | rs9325827 | 0.05 | 0.08 | IL4 | rs2243290 | 0.02 | 0.04 |
| NAT1 | rs4921880 | 0.52 | 0.02 | IL4R | rs3024530 | 0.03 | 0.05 |
| NFKB1 | rs10489113 | 0.39 | 0.04 | IL4R | rs3024537 | 0.06 | 0.02 |
| NFKBIE | rs2282151 | 0.02 | 0.06 | IL4R | rs3024547 | 0.06 | 0.02 |
| NR3C1 | rs33388 | 0.77 | 0.01 | IL4R | rs3024548 | 0.02 | 0.05 |
| NR3C1 | rs2918417 | 0.97 | 0.04 | IL4R | rs3024560 | 0.02 | 0.06 |
| NR3C1 | rs4634384 | 0.72 | 0.01 | IL4R | rs2239347 | 1.40E-03 | 3.80E-03 |
| PAFAH1B1 | rs7213463 | 0.02 | 0.06 | IL4R | rs3024676 | 0.03 | 0.08 |
| PGR | rs555572 | 0.01 | 0.03 | IL4R | rs1805015 | 0.04 | 0.1 |
| PGR | rs11224589 | 0.01 | 0.02 | MMP8 | rs1939020 | 0.03 | 0.06 |
| PGR | rs619487 | 0.04 | 0.02 | MTHFR | rs4846048 | 0.05 | 0.1 |
| PGRMC2 | rs11726595 | 0.11 | 4.7E-03 | MTHFR | rs1476413 | 0.05 | 0.02 |
| PLA2G4A | rs2076075 | 0.02 | 0.06 | MTHFR | rs1994798 | 3.90E-03 | 0.01 |
| PLAT | rs4581040 | 0.04 | 0.11 | MTHFR | rs17421462 | 0.01 | 0.01 |
| PLAT | rs2020922 | 0.03 | 0.08 | MTHFR | rs17421511 | 0.25 | 0.01 |
| PLG | rs4252092 | 0.01 | 0.03 | MTHFR | rs4846052 | 0.01 | 0.01 |
| PLG | rs783147 | 0.03 | 0.06 | MTHFR | rs11121832 | 0.01 | 0.02 |
| PLG | rs4252166 | 0.04 | 0.01 | MTHFR | rs9651118 | 2.50E-03 | 0.01 |
| PON1 | rs2299260 | 0.34 | 0.03 | MTHFR | rs3737964 | 0.01 | 0.02 |
| PON1 | rs854569 | 0.04 | 4.4E-03 | MTRR | rs1532268 | 0.88 | 0.05 |
| PON2 | rs43037 | 0.17 | 0.03 | NFKB1 | rs13117745 | 0.01 | 0.02 |
| PTGER3 | rs959 | 0.58 | 0.03 | NFKB1 | rs4648090 | 0.02 | 0.03 |
| PTGER3 | rs6685546 | 0.03 | 0.08 | NFKB1 | rs4648141 | 0.05 | 0.1 |
| PTGER3 | rs17131465 | 0.03 | 0.06 | NFKBIB | rs3136646 | 0.07 | 0.03 |
| PTGER3 | rs12119442 | 0.03 | 0.06 | NR3C1 | rs9324918 | 0.38 | 0.04 |
| PTGER3 | rs5702 | 0.57 | 0.02 | PGEA1 | rs6519132 | 0.05 | 0.05 |
| PTGER3 | rs7541092 | 0.03 | 0.03 | PGR | rs518162 | 0.01 | 0.02 |
| PTGER3 | rs1409165 | 0.02 | 0.03 | PGRMC1 | rs2428757 | 0.07 | 0.03 |
| PTGER3 | rs17131487 | 0.03 | 0.09 | PLAUR | rs4803648 | 0.95 | 0.01 |
| PTGER3 | rs875727 | 0.18 | 0.05 | PLAUR | rs4802189 | 0.72 | 0.01 |
| PTGER3 | rs977214 | 0.06 | 0.04 | PLAUR | rs4251854 | 0.21 | 0.03 |
| PTGER3 | rs6665776 | 0.06 | 0.04 | PLG | rs9458011 | 0.03 | 0.07 |
| PTGER3 | rs2072947 | 0.20 | 0.03 | PON1 | rs757158 | 0.01 | 0.01 |
| PTGES | rs2302821 | 0.05 | 0.01 | PON1 | rs854547 | 0.05 | 0.1 |
| PTGES | rs2241270 | 0.05 | 0.11 | PON1 | rs854548 | 2.50E-03 | 0.01 |
| PTGFR | rs3766345 | 0.03 | 0.08 | PON1 | rs854551 | 0.01 | 0.02 |
| PTGFR | rs668005 | 0.04 | 0.13 | PON1 | rs854552 | 1.80E-03 | 0.01 |
| SLC6A4 | rs4251417 | 0.01 | 0.01 | PON1 | rs2272365 | 0.04 | 0.12 |
| TFPI | rs12693471 | 0.01 | 0.01 | PON2 | rs2286233 | 0.04 | 0.11 |
| TFPI | rs8176541 | 0.01 | 0.01 | PTGER2 | rs1254600 | 0.11 | 0.04 |
| TFPI | rs7586970 | 0.01 | 0.01 | PTGER3 | rs17131465 | 0.05 | 0.04 |
| TFPI | rs3213739 | 0.03 | 2.4E-03 | PTGER3 | rs2256385 | 0.03 | 0.1 |
| TFPI | rs8176508 | 0.16 | 0.05 | PTGER3 | rs6424414 | 0.03 | 0.08 |
| TFPI | rs2041778 | 0.04 | 0.06 | PTGER3 | rs2300167 | 0.06 | 0.01 |
| TFPI | rs3755248 | 0.01 | 0.02 | PTGFR | rs1322934 | 0.11 | 0.02 |
| TFPI | rs7573488 | 0.01 | 0.01 | PTGFR | rs668005 | 0.02 | 0.01 |
| TIMP3 | rs2040435 | 0.83 | 0.05 | SERPINE1 | rs2227667 | 0.03 | 0.05 |
| TREM1 | rs16894387 | 0.04 | 0.12 | SHMT1 | rs1979277 | 0.02 | 0.01 |
| TREM1 | rs4711668 | 2.9E-03 | 0.01 | SLC6A4 | rs7224199 | 0.01 | 0.03 |
| TREM1 | rs6940092 | 0.02 | 0.05 | SLC6A4 | rs1042173 | 0.01 | 0.03 |
| TSHR | rs1035145 | 0.05 | 0.17 | SLC6A4 | rs3794808 | 0.02 | 0.06 |
| TSHR | rs2300520 | 0.04 | 0.08 | SLC6A4 | rs140701 | 0.02 | 0.07 |
| TSHR | rs2110697 | 0.40 | 0.04 | SLC6A4 | rs2020942 | 0.02 | 0.06 |
| TSHR | rs11845715 | 0.78 | 0.02 | SLC6A4 | rs4251417 | 8.00E-04 | 2.70E-03 |
| TSHR | rs3783938 | 0.05 | 0.17 | TCN2 | rs2267163 | 0.03 | 0.07 |
| UGT1A1 | rs4148329 | 0.43 | 0.03 | TFPI | rs12693471 | 4.00E-03 | 0.01 |
| UGT1A1 | rs6719561 | 0.07 | 0.01 | TFPI | rs8176541 | 4.30E-03 | 0.01 |
|  |  |  |  | TFPI | rs7586970 | 3.70E-03 | 0.01 |
|  |  |  |  | TFPI | rs3213739 | 4.40E-03 | 0.01 |
|  |  |  |  | TFPI | rs8176508 | 3.70E-03 | 0.01 |
|  |  |  |  | TFPI | rs2041778 | 0.03 | 0.08 |
|  |  |  |  | TFPI | rs3755248 | 0.03 | 0.09 |
|  |  |  |  | TFPI | rs7573488 | 0.02 | 0.06 |
|  |  |  |  | TFPI | rs6434222 | 5.80E-05* | 1.00E-04 |
|  |  |  |  | TIMP3 | rs5754289 | 0.87 | 0.03 |
|  |  |  |  | TIMP4 | rs3773364 | 0.29 | 0.04 |
|  |  |  |  | TLR4 | rs7869402 | 0.04 | 0.05 |
|  |  |  |  | TREM1 | rs6939973 | 0.02 | 0.04 |
|  |  |  |  | TSHR | rs4903964 | 0.44 | 0.03 |
|  |  |  |  | TSHR | rs3783943 | 0.05 | 0.14 |
|  |  |  |  | TSHR | rs930099 | 0.03 | 0.07 |
|  |  |  |  | UGT1A1 | rs929596 | 0.22 | 0.03 |
|  |  |  |  | UGT1A1 | rs2302538 | 0.86 | 0.04 |
|  |  |  |  | UGT1A1 | rs11888492 | 0.45 | 0.03 |

* Significant after correction for multiple testing with FDR (q=0.2)
