# Supplementary material for: Maternal and Fetal Genetic Associations of PTGER3 and PON1 with Preterm Birth
Source: PLoS One. 2010 Feb 3;5(2):e9040. doi: 10.1371/journal.pone.0009040 (PMC2815792; doi:10.1371/journal.pone.0009040)
Supplement: Table S4 — (0.39 MB DOC) [file pone.0009040.s006.doc]

Supplemental Table 4: Replication of maternal and fetal results.

| Population | Gene | SNP | Allele | Cenn Case MAF | Cenn Control MAF | Cenn Allele p | Cenn Genotype p | MoBa Case MAF | MoBa Control MAF | MoBa Allele p | MoBa Genotype p | Pooled Case MAF | Pooled Control MAF | Pooled Allele p | Pooled Genotype p |
| --- | --- | --- | --- | --- | --- | --- | --- | --- | --- | --- | --- | --- | --- | --- | --- |
| Maternal | ADRB2 | rs1432622 | T | 0.47 | 0.42 | 0.195 | 0.264 | 0.45 | 0.38 | 0.048 | 0.128 | 0.46 | 0.40 | 0.023* | 0.048* |
|  | ADRB2 | rs12654778 | A | 0.33 | 0.40 | 0.083 | 0.129 | 0.37 | 0.45 | 0.028 | 0.088 | 0.36 | 0.42 | 0.008* | 0.019* |
|  | AP3M2 | rs4581040 | G | 0.32 | 0.24 | 0.042 | 0.131 | 0.30 | 0.24 | 0.041 | 0.112 | 0.31 | 0.24 | 0.004* | 0.017* |
|  | CBS | rs6586282 | T | 0.19 | 0.19 | 0.837 | 0.165 | 0.18 | 0.15 | 0.219 | 0.005 | 0.19 | 0.17 | 0.330 | 0.585 |
|  | CBS | rs11203172 | T | 0.22 | 0.16 | 0.092 | 0.176 | 0.17 | 0.20 | 0.318 | 0.049 | 0.19 | 0.18 | 0.714 | 0.591 |
|  | CCL2 | rs1024610 | A | 0.21 | 0.20 | 0.657 | 0.007 | 0.18 | 0.15 | 0.184 | 0.180 | 0.19 | 0.17 | 0.258 | 0.185 |
|  | COL1A1 | rs1061237 | C | 0.23 | 0.28 | 0.090 | 0.055 | 0.26 | 0.29 | 0.332 | 0.044 | 0.24 | 0.29 | 0.069* | 0.003* |
|  | COL1A2 | rs420257 | C | 0.36 | 0.29 | 0.042 | 0.128 | 0.23 | 0.29 | 0.064 | 0.111 | 0.29 | 0.29 | 0.924 | 0.637 |
|  | COL1A2 | rs389328 | T | 0.20 | 0.13 | 0.031 | 0.083 | 0.12 | 0.16 | 0.131 | 0.334 | 0.15 | 0.15 | 0.760 | 0.851 |
|  | COL1A2 | rs2521205 | G | 0.57 | 0.47 | 0.015 | 0.066 | 0.43 | 0.51 | 0.021 | 0.045 | 0.49 | 0.49 | 0.832 | 0.758 |
|  | COL1A2 | rs7804898 | G | 0.08 | 0.12 | 0.050 | 0.105 | 0.17 | 0.14 | 0.214 | 0.015 | 0.13 | 0.13 | 0.984 | 0.059* |
|  | COL3A1 | rs2271682 | A | 0.25 | 0.29 | 0.195 | 0.181 | 0.25 | 0.31 | 0.040 | 0.053 | 0.25 | 0.30 | 0.018* | 0.011* |
|  | COL5A1 | rs12005720 | C | 0.11 | 0.17 | 0.032 | 0.091 | 0.19 | 0.16 | 0.284 | 0.041 | 0.16 | 0.17 | 0.681 | 0.122 |
|  | COL5A1 | rs4842167 | C | 0.48 | 0.38 | 0.009 | 0.021 | 0.36 | 0.44 | 0.011 | 0.037 | 0.41 | 0.41 | 0.845 | 0.526 |
|  | COL5A1 | rs3811161 | C | 0.43 | 0.51 | 0.028 | 0.087 | 0.53 | 0.44 | 0.012 | 0.044 | 0.49 | 0.48 | 0.646 | 0.897 |
|  | COL5A1 | rs10745387 | A | 0.46 | 0.39 | 0.110 | 0.150 | 0.37 | 0.44 | 0.031 | 0.101 | 0.41 | 0.42 | 0.569 | 0.596 |
|  | CRHBP | rs10055255 | A | 0.33 | 0.45 | 0.001 | 0.006 | 0.41 | 0.36 | 0.125 | 0.286 | 0.38 | 0.40 | 0.274 | 0.548 |
|  | CRHBP | rs1875999 | G | 0.29 | 0.40 | 0.005 | 0.023 | 0.38 | 0.33 | 0.113 | 0.250 | 0.34 | 0.36 | 0.485 | 0.784 |
|  | CRHR2 | rs12701020 | T | 0.16 | 0.15 | 0.551 | 0.191 | 0.18 | 0.14 | 0.086 | 0.015 | 0.17 | 0.14 | 0.090* | 0.006* |
|  | CYP19A1 | rs17703982 | T | 0.09 | 0.06 | 0.141 | 0.037 | 0.07 | 0.04 | 0.060 | 0.086 | 0.07 | 0.05 | 0.023* | 0.017* |
|  | F5 | rs2187952 | A | 0.24 | 0.31 | 0.045 | 0.098 | 0.24 | 0.29 | 0.126 | 0.242 | 0.24 | 0.30 | 0.012* | 0.026* |
|  | IL1B | rs1143630 | A | 0.09 | 0.07 | 0.436 | 0.168 | 0.04 | 0.08 | 0.025 | 0.044 | 0.06 | 0.08 | 0.263 | 0.131 |
|  | IL1R1 | rs3917273 | T | 0.39 | 0.47 | 0.034 | 0.092 | 0.42 | 0.35 | 0.036 | 0.015 | 0.41 | 0.41 | 1.000 | 0.396 |
|  | IL1R1 | rs2110726 | T | 0.44 | 0.34 | 0.007 | 0.014 | 0.40 | 0.43 | 0.425 | 0.026 | 0.42 | 0.38 | 0.216 | 0.211 |
|  | IL1R2 | rs4851520 | C | 0.15 | 0.10 | 0.046 | 0.103 | 0.15 | 0.11 | 0.059 | 0.162 | 0.15 | 0.11 | 0.006* | 0.016* |
|  | IL1R2 | rs4851522 | T | 0.16 | 0.11 | 0.042 | 0.072 | 0.15 | 0.11 | 0.090 | 0.233 | 0.16 | 0.11 | 0.009* | 0.022* |
|  | IL1R2 | rs1108338 | C | 0.28 | 0.21 | 0.038 | 0.082 | 0.29 | 0.23 | 0.022 | 0.012 | 0.29 | 0.22 | 0.002* | 0.001* |
|  | IL1RAP | rs7628333 | T | 0.21 | 0.24 | 0.358 | 0.080 | 0.22 | 0.32 | 0.002 | 0.005 | 0.22 | 0.28 | 0.004* | 0.012* |
|  | IL2RA | rs2031229 | A | 0.19 | 0.26 | 0.036 | 0.142 | 0.28 | 0.23 | 0.088 | 0.074 | 0.25 | 0.25 | 0.999 | 0.408 |
|  | IL2RA | rs1107345 | A | 0.21 | 0.25 | 0.198 | 0.471 | 0.28 | 0.22 | 0.042 | 0.063 | 0.25 | 0.23 | 0.464 | 0.443 |
|  | IL2RA | rs11256497 | A | 0.32 | 0.37 | 0.167 | 0.314 | 0.31 | 0.38 | 0.035 | 0.104 | 0.31 | 0.37 | 0.012* | 0.045* |
|  | IL4R | rs3024530 | G | 0.52 | 0.44 | 0.025 | 0.062 | 0.47 | 0.42 | 0.157 | 0.061 | 0.49 | 0.43 | 0.014* | 0.010* |
|  | IL4R | rs3024548 | C | 0.51 | 0.43 | 0.039 | 0.075 | 0.46 | 0.41 | 0.132 | 0.038 | 0.48 | 0.42 | 0.015* | 0.006* |
|  | IL6R | rs4845374 | T | 0.13 | 0.18 | 0.139 | 0.110 | 0.12 | 0.17 | 0.042 | 0.027 | 0.13 | 0.17 | 0.012* | 0.004* |
|  | IL6R | rs4329505 | C | 0.13 | 0.17 | 0.129 | 0.067 | 0.12 | 0.17 | 0.042 | 0.027 | 0.13 | 0.17 | 0.011* | 0.003* |
|  | MMP2 | rs1053605 | T | 0.04 | 0.06 | 0.184 | 0.304 | 0.09 | 0.05 | 0.037 | 0.068 | 0.07 | 0.06 | 0.383 | 0.482 |
|  | MTRR | rs162031 | T | 0.28 | 0.23 | 0.160 | 0.303 | 0.21 | 0.28 | 0.016 | 0.054 | 0.23 | 0.25 | 0.331 | 0.413 |
|  | NAT1 | rs7017402 | A | 0.15 | 0.10 | 0.038 | 0.088 | 0.13 | 0.09 | 0.043 | 0.048 | 0.14 | 0.09 | 0.004* | 0.013* |
|  | NAT1 | rs9325827 | C | 0.18 | 0.11 | 0.012 | 0.040 | 0.16 | 0.11 | 0.048 | 0.083 | 0.17 | 0.11 | 0.002* | 0.008* |
|  | NAT1 | rs4921880 | T | 0.28 | 0.23 | 0.147 | 0.055 | 0.27 | 0.25 | 0.523 | 0.021 | 0.27 | 0.24 | 0.149 | 0.290 |
|  | PGR | rs555572 | C | 0.30 | 0.34 | 0.243 | 0.130 | 0.35 | 0.27 | 0.007 | 0.026 | 0.33 | 0.30 | 0.235 | 0.135 |
|  | PGR | rs11224589 | C | 0.30 | 0.35 | 0.210 | 0.138 | 0.37 | 0.28 | 0.007 | 0.021 | 0.34 | 0.31 | 0.253 | 0.125 |
|  | PGR | rs619487 | G | 0.37 | 0.31 | 0.102 | 0.132 | 0.33 | 0.39 | 0.040 | 0.018 | 0.34 | 0.35 | 0.682 | 0.065* |
|  | PLA2G4A | rs2076075 | A | 0.14 | 0.10 | 0.110 | 0.236 | 0.17 | 0.12 | 0.020 | 0.056 | 0.16 | 0.11 | 0.004* | 0.016* |
|  | PLAT | rs2020922 | A | 0.32 | 0.24 | 0.029 | 0.095 | 0.30 | 0.24 | 0.034 | 0.081 | 0.31 | 0.24 | 0.003* | 0.009* |
|  | PLG | rs783147 | T | 0.40 | 0.46 | 0.139 | 0.117 | 0.44 | 0.52 | 0.026 | 0.060 | 0.43 | 0.49 | 0.012* | 0.010* |
|  | PTGER3 | rs959 | G | 0.25 | 0.25 | 0.858 | 0.055 | 0.25 | 0.24 | 0.581 | 0.035 | 0.25 | 0.24 | 0.780 | 0.955 |
|  | PTGER3 | rs5702 | T | 0.24 | 0.25 | 0.681 | 0.154 | 0.27 | 0.25 | 0.575 | 0.020 | 0.25 | 0.25 | 0.852 | 0.862 |
|  | PTGER3 | rs1409165 | C | 0.14 | 0.11 | 0.169 | 0.432 | 0.09 | 0.14 | 0.024 | 0.031 | 0.11 | 0.12 | 0.443 | 0.763 |
|  | PTGER3 | rs977214 | G | 0.09 | 0.13 | 0.166 | 0.005 | 0.06 | 0.09 | 0.064 | 0.039 | 0.07 | 0.11 | 0.016* | 3x10^-4^** |
|  | PTGER3 | rs6665776 | A | 0.09 | 0.12 | 0.235 | 0.010 | 0.06 | 0.09 | 0.064 | 0.039 | 0.07 | 0.11 | 0.024* | 5x10^-4^* |
|  | PTGER3 | rs2072947 | C | 0.53 | 0.44 | 0.017 | 0.034 | 0.41 | 0.46 | 0.205 | 0.027 | 0.46 | 0.45 | 0.582 | 0.039* |
|  | TFPI | rs3755248 | G | 0.36 | 0.31 | 0.197 | 0.344 | 0.27 | 0.35 | 6.5x10^-3^ | 0.02 | 0.30 | 0.34 | 0.180 | 0.387 |
|  | TIMP3 | rs130290 | T | 0.06 | 0.11 | 0.013 | 0.022 | 0.08 | 0.05 | 0.151 | 0.234 | 0.07 | 0.08 | 0.373 | 0.644 |
|  | TIMP3 | rs130293 | C | 0.05 | 0.11 | 0.011 | 0.025 | 0.08 | 0.05 | 0.150 | 0.228 | 0.07 | 0.08 | 0.378 | 0.636 |
|  | TLR2 | rs1898830 | G | 0.29 | 0.38 | 0.015 | 0.052 | 0.33 | 0.38 | 0.152 | 0.345 | 0.31 | 0.38 | 0.008* | 0.025* |
|  | TNFRSF1A | rs4149578 | A | 0.12 | 0.07 | 0.033 | 0.044 | 0.14 | 0.10 | 0.156 | 0.314 | 0.13 | 0.09 | 0.012* | 0.028* |
|  | TSHR | rs11845715 | T | 0.22 | 0.15 | 0.025 | 0.099 | 0.17 | 0.17 | 0.779 | 0.023 | 0.19 | 0.16 | 0.093* | 0.025* |
|  | TSHR | rs17630128 | C | 0.33 | 0.25 | 0.031 | 0.023 | 0.36 | 0.30 | 0.072 | 0.192 | 0.35 | 0.28 | 0.004* | 0.010* |
|  | TSHR | rs12883801 | G | 0.41 | 0.52 | 0.005 | 0.011 | 0.37 | 0.43 | 0.107 | 0.137 | 0.39 | 0.47 | 0.001* | 0.001* |
| Fetal | C6orf48 | rs2471980 | G | 0.36 | 0.31 | 0.239 | 0.170 | 0.28 | 0.36 | 0.019 | 0.004 | 0.31 | 0.34 | 0.316 | 0.458 |
|  | COL1A2 | rs42524 | G | 0.23 | 0.21 | 0.440 | 0.055 | 0.19 | 0.26 | 0.010 | 0.022 | 0.21 | 0.24 | 0.154 | 0.023* |
|  | COL1A2 | rs42528 | T | 0.24 | 0.23 | 0.699 | 0.135 | 0.20 | 0.28 | 0.008 | 0.018 | 0.21 | 0.25 | 0.079* | 0.025* |
|  | COL3A1 | rs2271682 | A | 0.24 | 0.29 | 0.120 | 0.045 | 0.26 | 0.32 | 0.062 | 0.108 | 0.25 | 0.31 | 0.017* | 0.009* |
|  | COL3A1 | rs3134656 | A | 0.50 | 0.39 | 0.005 | 0.026 | 0.47 | 0.41 | 0.067 | 0.098 | 0.48 | 0.40 | 0.001* | 0.004* |
|  | COL5A2 | rs9288163 | A | 0.11 | 0.06 | 0.025 | 0.034 | 0.08 | 0.09 | 0.467 | 0.192 | 0.09 | 0.08 | 0.361 | 0.057* |
|  | EDN2 | rs4660541 | T | 0.24 | 0.28 | 0.254 | 0.012 | 0.27 | 0.28 | 0.648 | 0.159 | 0.26 | 0.28 | 0.287 | 0.538 |
|  | EPHX2 | rs10503812 | A | 0.21 | 0.15 | 0.040 | 0.118 | 0.15 | 0.13 | 0.574 | 0.029 | 0.17 | 0.14 | 0.082* | 0.025* |
|  | EPHX2 | rs4149239 | G | 0.21 | 0.15 | 0.047 | 0.110 | 0.15 | 0.13 | 0.575 | 0.028 | 0.17 | 0.14 | 0.092* | 0.017* |
|  | EPHX2 | rs4149252 | T | 0.20 | 0.15 | 0.052 | 0.153 | 0.15 | 0.13 | 0.575 | 0.028 | 0.17 | 0.14 | 0.097* | 0.029* |
|  | EPHX2 | rs4149259 | T | 0.21 | 0.14 | 0.024 | 0.053 | 0.15 | 0.13 | 0.420 | 0.015 | 0.17 | 0.14 | 0.040* | 0.005* |
|  | EPHX2 | rs891401 | G | 0.21 | 0.15 | 0.030 | 0.081 | 0.15 | 0.13 | 0.574 | 0.029 | 0.17 | 0.14 | 0.070* | 0.025* |
|  | HSPA6 | rs9427401 | C | 0.14 | 0.11 | 0.160 | 0.381 | 0.15 | 0.09 | 0.007 | 0.022 | 0.14 | 0.10 | 0.004* | 0.013* |
|  | IL1A | rs17561 | T | 0.24 | 0.33 | 0.012 | 0.043 | 0.35 | 0.28 | 0.025 | 0.058 | 0.30 | 0.30 | 0.887 | 0.891 |
|  | IL1A | rs1878321 | C | 0.24 | 0.33 | 0.014 | 0.056 | 0.35 | 0.28 | 0.025 | 0.058 | 0.31 | 0.30 | 0.875 | 0.814 |
|  | IL1A | rs2856838 | T | 0.42 | 0.35 | 0.073 | 0.158 | 0.32 | 0.40 | 0.027 | 0.023 | 0.36 | 0.38 | 0.607 | 0.547 |
|  | IL1B | rs1143630 | A | 0.10 | 0.05 | 0.036 | 0.025 | 0.05 | 0.08 | 0.151 | 0.147 | 0.07 | 0.07 | 0.740 | 0.747 |
|  | IL1R1 | rs3917225 | G | 0.47 | 0.41 | 0.169 | 0.351 | 0.45 | 0.53 | 0.031 | 0.083 | 0.46 | 0.47 | 0.515 | 0.799 |
|  | IL1RAP | rs9845825 | A | 0.40 | 0.30 | 0.015 | 0.011 | 0.35 | 0.30 | 0.132 | 0.311 | 0.37 | 0.30 | 0.007* | 0.018* |
|  | IL1RN | rs315920 | T | 0.24 | 0.21 | 0.276 | 0.040 | 0.18 | 0.22 | 0.198 | 0.108 | 0.21 | 0.21 | 0.783 | 0.013* |
|  | IL2RA | rs12722596 | G | 0.08 | 0.13 | 0.026 | 0.084 | 0.13 | 0.08 | 0.035 | 0.081 | 0.11 | 0.11 | 0.924 | 0.847 |
|  | IL4R | rs1805015 | C | 0.23 | 0.18 | 0.096 | 0.228 | 0.16 | 0.11 | 0.039 | 0.103 | 0.19 | 0.14 | 0.013* | 0.044* |
|  | IL4R | rs3024676 | A | 0.24 | 0.19 | 0.099 | 0.149 | 0.17 | 0.11 | 0.031 | 0.076 | 0.20 | 0.15 | 0.012* | 0.038* |
|  | MMP1 | rs1155764 | G | 0.20 | 0.12 | 0.011 | 0.019 | 0.11 | 0.15 | 0.109 | 0.091 | 0.15 | 0.14 | 0.618 | 0.558 |
|  | MTHFR | rs1476413 | A | 0.26 | 0.31 | 0.197 | 0.427 | 0.25 | 0.31 | 0.049 | 0.021 | 0.26 | 0.31 | 0.019* | 0.026* |
|  | MTHFR | rs1994798 | C | 0.39 | 0.45 | 0.131 | 0.281 | 0.37 | 0.47 | 0.004 | 0.013 | 0.38 | 0.46 | 0.001* | 0.006* |
|  | MTHFR | rs4846048 | G | 0.28 | 0.33 | 0.189 | 0.352 | 0.25 | 0.31 | 0.047 | 0.101 | 0.26 | 0.32 | 0.016* | 0.054* |
|  | MTHFR | rs4846052 | T | 0.39 | 0.46 | 0.085 | 0.205 | 0.36 | 0.45 | 0.009 | 0.010 | 0.37 | 0.46 | 0.002* | 0.003* |
|  | NFKB1 | rs13117745 | T | 0.18 | 0.14 | 0.149 | 0.375 | 0.17 | 0.11 | 0.011 | 0.019 | 0.18 | 0.12 | 0.005* | 0.016* |
|  | NFKB1 | rs4648141 | A | 0.20 | 0.15 | 0.067 | 0.140 | 0.19 | 0.14 | 0.047 | 0.102 | 0.19 | 0.14 | 0.007* | 0.026* |
|  | NR3C1 | rs9324918 | G | 0.21 | 0.14 | 0.031 | 0.076 | 0.21 | 0.18 | 0.380 | 0.036 | 0.21 | 0.16 | 0.036* | 0.005* |
|  | PGR | rs11224589 | C | 0.26 | 0.34 | 0.024 | 0.078 | 0.34 | 0.28 | 0.085 | 0.231 | 0.31 | 0.31 | 0.875 | 0.752 |
|  | PGR | rs555572 | C | 0.26 | 0.34 | 0.023 | 0.055 | 0.33 | 0.28 | 0.117 | 0.294 | 0.30 | 0.31 | 0.755 | 0.690 |
|  | PON1 | rs2272365 | G | 0.19 | 0.13 | 0.046 | 0.052 | 0.17 | 0.12 | 0.041 | 0.121 | 0.18 | 0.13 | 0.005* | 0.014* |
|  | PON1 | rs854552 | C | 0.22 | 0.28 | 0.120 | 0.248 | 0.21 | 0.30 | 0.002 | 0.006 | 0.21 | 0.29 | 8x10^-4^* | 0.002* |
|  | PON2 | rs2286233 | A | 0.09 | 0.16 | 0.009 | 0.041 | 0.10 | 0.15 | 0.037 | 0.106 | 0.09 | 0.15 | 9x10^-4^* | 0.005* |
|  | PTGER2 | rs1254600 | T | 0.15 | 0.20 | 0.131 | 0.157 | 0.20 | 0.16 | 0.112 | 0.042 | 0.18 | 0.18 | 0.825 | 0.089* |
|  | SLC6A4 | rs1042173 | G | 0.40 | 0.47 | 0.093 | 0.211 | 0.40 | 0.49 | 0.008 | 0.032 | 0.40 | 0.48 | 0.002* | 0.008* |
|  | SLC6A4 | rs140701 | A | 0.38 | 0.43 | 0.159 | 0.322 | 0.39 | 0.47 | 0.022 | 0.074 | 0.38 | 0.45 | 0.009* | 0.028* |
|  | SLC6A4 | rs3794808 | A | 0.37 | 0.45 | 0.044 | 0.109 | 0.39 | 0.47 | 0.018 | 0.062 | 0.38 | 0.46 | 0.002* | 0.007* |
|  | SLC6A4 | rs7224199 | T | 0.42 | 0.47 | 0.175 | 0.359 | 0.40 | 0.49 | 0.008 | 0.032 | 0.41 | 0.48 | 0.004* | 0.013* |
|  | TREM1 | rs4711668 | T | 0.27 | 0.36 | 0.017 | 0.030 | 0.34 | 0.28 | 0.072 | 0.134 | 0.31 | 0.32 | 0.818 | 0.382 |
|  | TSHR | rs179247 | G | 0.51 | 0.42 | 0.024 | 0.024 | 0.42 | 0.46 | 0.174 | 0.141 | 0.46 | 0.45 | 0.680 | 0.121* |
|  | UGT1A1 | rs11888492 | C | 0.15 | 0.10 | 0.044 | 0.117 | 0.13 | 0.11 | 0.449 | 0.033 | 0.14 | 0.10 | 0.060* | 0.131* |

* Significant after correction for multiple testing with FDR (q=0.2)

** Significant after correction for multiple testing with Bonferroni
