# Supplementary material for: Maternal and Fetal Genetic Associations of PTGER3 and PON1 with Preterm Birth
Source: PLoS One. 2010 Feb 3;5(2):e9040. doi: 10.1371/journal.pone.0009040 (PMC2815792; doi:10.1371/journal.pone.0009040)
Supplement: Table S5 — (0.20 MB RTF) [file pone.0009040.s007.rtf]

Supplemental Table5: Global p-values for haplotype Associations in Cenn and Moba studies for a) maternal PTGER3 and b) fetal PON1
Haplotype	Cenn	Moba	
rs959rs1327460rs6656853rs7530345	0.480	0.852	
rs959rs1327460	0.462	0.902	
rs959rs1327460rs6656853	0.493	0.810	
rs1327460rs6656853rs7530345rs6685546	0.715	0.008	
rs1327460rs6656853rs7530345	0.559	0.868	
rs1327460rs6656853	0.526	0.853	
rs6656853rs7530345rs6685546rs17131465	0.673	0.023	
rs6656853rs7530345	0.489	0.919	
rs6656853rs7530345rs6685546	0.646	0.008	
rs7530345rs6685546	0.761	0.037	
rs7530345rs6685546rs17131465rs12119442	0.690	0.026	
rs7530345rs6685546rs17131465	0.690	0.026	
rs6685546rs17131465rs12119442rs5702	0.723	0.031	
rs6685546rs17131465	0.517	0.015	
rs6685546rs17131465rs12119442	0.517	0.015	
rs17131465rs12119442	0.274	0.026	
rs17131465rs12119442rs5702	0.541	0.085	
rs17131465rs12119442rs5702rs1409985	0.536	0.066	
rs12119442rs5702	0.541	0.085	
rs12119442rs5702rs1409985	0.536	0.066	
rs12119442rs5702rs1409985rs1327449	0.754	0.129	
rs5702rs1409985rs1327449rs4649932	0.600	0.038	
rs5702rs1409985	0.573	0.021	
rs5702rs1409985rs1327449	0.787	0.047	
rs1409985rs1327449	0.897	0.277	
rs1409985rs1327449rs4649932	0.630	0.255	
rs1409985rs1327449rs4649932rs1409981	0.500	0.001	
rs1327449rs4649932rs1409981rs4147115	0.624	0.040	
rs1327449rs4649932rs1409981	0.422	0.001	
rs1327449rs4649932	0.870	0.539	
rs4649932rs1409981rs4147115rs1409165	0.371	0.352	
rs4649932rs1409981	0.493	0.030	
rs4649932rs1409981rs4147115	0.707	0.337	
rs1409981rs4147115rs1409165	0.246	0.352	
rs1409981rs4147115rs1409165rs4650094	0.252	0.372	
rs1409981rs4147115	0.732	0.414	
rs4147115rs1409165rs4650094	0.251	0.257	
rs4147115rs1409165	0.158	0.064	
rs4147115rs1409165rs4650094rs875727	0.316	0.368	
rs1409165rs4650094rs875727	0.248	0.075	
rs1409165rs4650094rs875727rs17541722	0.236	0.133	
rs1409165rs4650094	0.593	0.100	
rs4650094rs875727	0.435	0.403	
rs4650094rs875727rs17541722rs1327466	0.239	0.280	
rs4650094rs875727rs17541722	0.584	0.254	
rs875727rs17541722	0.420	0.198	
rs875727rs17541722rs1327466rs1887404	0.539	0.197	
rs875727rs17541722rs1327466	0.444	0.303	
rs17541722rs1327466rs1887404rs17542063	0.298	0.415	
rs17541722rs1327466rs1887404	0.748	0.313	
rs17541722rs1327466	0.808	0.345	
rs1327466rs1887404rs17542063	0.267	0.390	
rs1327466rs1887404	0.625	0.291	
rs1327466rs1887404rs17542063rs6424410	0.424	0.420	
rs1887404rs17542063	0.258	0.435	
rs1887404rs17542063rs6424410rs602383	0.122	0.216	
rs1887404rs17542063rs6424410	0.344	0.476	
rs17542063rs6424410	0.292	0.572	
rs17542063rs6424410rs602383rs578096	0.089	0.370	
rs17542063rs6424410rs602383	0.177	0.240	
rs6424410rs602383rs578096rs6670616	0.023	0.376	
rs6424410rs602383	0.108	0.723	
rs6424410rs602383rs578096	0.171	0.645	
rs602383rs578096	0.063	0.934	
rs602383rs578096rs6670616	0.019	0.451	
rs602383rs578096rs6670616rs2421735	0.015	0.376	
rs578096rs6670616	0.004	0.956	
rs578096rs6670616rs2421735	0.003	0.985	
rs578096rs6670616rs2421735rs977214	0.007	0.283	
rs6670616rs2421735rs977214rs6665776	0.057	0.172	
rs6670616rs2421735rs977214	0.049	0.166	
rs6670616rs2421735	0.039	0.980	
rs2421735rs977214rs6665776	0.062	0.166	
rs2421735rs977214	0.055	0.166	
rs2421735rs977214rs6665776rs594454	0.007	0.323	
rs977214rs6665776	0.223	0.063	
rs977214rs6665776rs594454rs2300161	0.008	0.261	
rs977214rs6665776rs594454	0.003	0.139	
rs6665776rs594454rs2300161	0.011	0.261	
rs6665776rs594454rs2300161rs5697	0.011	0.261	
rs6665776rs594454	0.004	0.139	
rs594454rs2300161	0.004	0.935	
rs594454rs2300161rs5697	0.004	0.935	
rs594454rs2300161rs5697rs2072947	0.009	0.261	
rs2300161rs5697rs2072947rs481940	0.057	0.252	
rs2300161rs5697	0.033	0.997	
rs2300161rs5697rs2072947	0.030	0.307	
rs5697rs2072947rs481940rs3819783	0.062	0.250	
rs5697rs2072947	0.032	0.307	
rs5697rs2072947rs481940	0.056	0.252	
rs2072947rs481940rs3819783rs3819790	0.059	0.246	
rs2072947rs481940rs3819783	0.059	0.246	
rs2072947rs481940	0.059	0.246	
rs481940rs3819783rs3819790	0.068	0.246	
rs481940rs3819783rs3819790rs726764	0.122	0.141	
rs481940rs3819783	0.068	0.246	
rs3819783rs3819790	0.129	0.901	
rs3819783rs3819790rs726764	0.156	0.169	
rs3819783rs3819790rs726764rs1409164	0.258	0.277	
rs3819790rs726764	0.156	0.169	
rs3819790rs726764rs1409164rs2256385	0.212	0.285	
rs3819790rs726764rs1409164	0.258	0.277	
rs726764rs1409164	0.762	0.202	
rs726764rs1409164rs2256385	0.781	0.188	
rs726764rs1409164rs2256385rs2300164	0.157	0.311	
rs1409164rs2256385	0.846	0.195	
rs1409164rs2256385rs2300164rs2050066	0.058	0.412	
rs1409164rs2256385rs2300164	0.177	0.324	
rs2256385rs2300164	0.094	0.318	
rs2256385rs2300164rs2050066rs6424414	0.079	0.303	
rs2256385rs2300164rs2050066	0.074	0.303	
rs2300164rs2050066	0.135	0.234	
rs2300164rs2050066rs6424414rs2300167	0.034	0.431	
rs2300164rs2050066rs6424414	0.084	0.297	
rs2050066rs6424414rs2300167rs6678886	0.048	0.476	
rs2050066rs6424414rs2300167	0.017	0.296	
rs2050066rs6424414	0.336	0.177	
rs6424414rs2300167	0.018	0.323	
rs6424414rs2300167rs6678886rs10789314	0.051	0.183	
rs6424414rs2300167rs6678886	0.098	0.280	
rs2300167rs6678886rs10789314rs5693	0.024	0.358	
rs2300167rs6678886rs10789314	0.086	0.527	
rs2300167rs6678886	0.060	0.350	
rs6678886rs10789314	0.182	0.750	
rs6678886rs10789314rs5693rs5680	0.007	0.805	
rs6678886rs10789314rs5693	0.010	0.807	
rs10789314rs5693	0.018	0.642	
rs10789314rs5693rs5680	0.006	0.805	
rs10789314rs5693rs5680rs5673	0.015	0.509	
rs5693rs5680rs5673rs11209736	0.036	0.637	
rs5693rs5680	0.035	0.688	
rs5693rs5680rs5673	0.066	0.429	
rs5680rs5673rs11209736rs8179390	0.012	0.566	
rs5680rs5673	0.032	0.361	
rs5680rs5673rs11209736	0.020	0.611	
rs5673rs11209736	0.015	0.348	
rs5673rs11209736rs8179390rs2817864	0.008	0.565	
rs5673rs11209736rs8179390	0.009	0.545	
rs11209736rs8179390rs2817864rs3000466	0.082	0.723	
rs11209736rs8179390rs2817864	0.027	0.530	
rs11209736rs8179390	0.028	0.501	
rs8179390rs2817864rs3000466	0.073	0.851	
rs8179390rs2817864	0.024	0.709	
rs2817864rs3000466	0.077	0.791	
